# STIP1 drives Metabolic Reprogramming in Esophageal Squamous Cell Carcinoma via AHCY‐LDHA Axis

**DOI:** 10.1002/EXP.20240198

**Published:** 2025-05-25

**Authors:** Guoguo Jin, Yanming Song, Mingyang Yan, Shaobo Fang, Yang Shao, Kexin Zhao, Meng Liu, Qinxin Guo, Xinyang Jia, Chengjuan Zhang, Zhenwei Wang, Kangdong Liu, Xiang Li, Simin Zhao, Mee‐Hyun Lee, Zhiping Guo, Zigang Dong

**Affiliations:** ^1^ Henan Key Laboratory of Chronic Disease Management Fuwai Central China Cardiovascular Hospital Zhengzhou China; ^2^ China‐US (Henan) Hormel Cancer Institute Zhengzhou Henan China; ^3^ Central China Subcenter of National Center for Cardiovascular Diseases Henan Cardiovascular Disease Center Fuwai Central‐China Cardiovascular Hospital Central China Fuwai Hospital of Zhengzhou University Zhengzhou China; ^4^ Tianjian Laboratory of Advanced Biomedical Sciences Institute of Advanced Biomedical Sciences Zhengzhou University Zhengzhou Henan China; ^5^ Department of Pathophysiology School of Basic Medical Sciences Zhengzhou University Zhengzhou Henan China; ^6^ Department of Medical Imaging Zhengzhou University People's Hospital& Henan Provincial People's Hospital Zhengzhou China; ^7^ Center of Bio Repository The Affiliated Cancer Hospital of Zhengzhou University Zhengzhou China; ^8^ Affiliated Cancer Hospital of Zhengzhou University Zhengzhou Henan P.R. China; ^9^ College of Korean Medicine Dongshin University Naju Republic of Korea

**Keywords:** AHCY, ESCC, glycolysis, heat stimulation, STIP1

## Abstract

Glucose metabolism reprogramming has emerged as a hallmark of cancer. We have reported that high temperature food or drink (>65°C) is the key etiological factors contributing to esophageal squamous cell carcinoma (ESCC) progression. Intriguingly, we observed that heat stimulation (42°C) alters glycolytic pathways in esophagus cells, but the underlying mechanisms remain poorly understood. Our findings revealed that stress‐induced phosphoprotein 1 (STIP1) exhibits elevated expression in esophageal tissues exposed to heat stimulation (>65°C) compared to unexposed tissues, and its overexpression correlated with clinical grade and predict poor prognosis in ESCC patients. Mechanistically, STIP1 interacts with and activates adenosylhomocysteinase (AHCY; also termed SAHH) and change the conformation of AHCY. STIP1 also facilitates AHCY binding to lactate dehydrogenase A (LDHA), stimulating glycolysis. Notably, AHCY recruits protein arginine methyltransferase 3 (PRMT3) to methylate LDHA at R106, inhibiting ubiquitination‐mediated AHCY degradation. In vivo, STIP1 knockout in mice dramatically inhibits 4‐nitrochinoline‐oxide (4NQO) induced esophageal tumorigenesis. Through virtual screening and functional validation, we identified licochalcone A (LCA) as a potent inhibitor of STIP1‐driven ESCC proliferation in vitro and in vivo. In summary, these findings delineate a pro‐tumorigenic signaling pathway whereby heat‐induced STIP1 upregulation promotes ESCC glycolysis and growth via moonlighting functions that coordinate AHCY activity and LDHA methylation.

## Graphical abstract

A schematic model illustrating that LCA can inhibit ESCC growth by inhibiting the STIP1/AHCY/LDHA axis. In brief. Jin et al. demonstrated that targeting STIP1 can significantly inhibit the progression of esophageal squamous cell carcinoma. The high expression of STIP1 increased the interaction between AHCY and LDHA and then AHCY recruits PRMT3 to methylate LDHA at R106 to promote the proliferation of cancer cells. LCA can inhibit the STIP1/AHCY/LDHA axis and inhibit tumor development mediated by the gly.

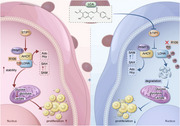

## 1 Introduction

Esophageal cancer represented a substantial health burden in 2020, accounting for at least 600,000 new cases and 540,000 deaths worldwide [1], positioning it as the sixth leading cause of cancer mortality [2]. ESCC constitutes the predominant histological subtype of esophageal malignancy, with over half of cases occurring in China [3]. Although neoadjuvant chemoradiotherapy followed by surgical resection is a primary therapeutic approach for locoregional ESCC, approximately 40% of patients experience recurrence and treatment failure, yielding poor 5‐year survival rates [4]. Therefore, elucidating the molecular and pathogenic mechanisms underlying ESCC oncogenesis is imperative.

Our group propose the cancer etiology and prevention principle “1+X”, where 1denotes the primary risk factor for a cancer and X represents the secondary contributing risk factors for cancer [5]. We reported that heat stimulation (>65°C) is the main risk factor of ESCC. Our previous data showed that drinking 65°C water can promote ESCC tumor progression from preneoplastic lesions to ESCC [6]. Interestingly, our findings have elucidated that heat stimulation can induce alterations in glycolytic pathways, although the underlying molecular mechanisms remain insufficiently delineated.

STIP1, also termed Hop, is a eukaryotic co‐chaperone that cooperates with Hsp70 and Hsp90 to facilitate client protein transfer [7]. Emerging evidence indicates that aberrant STIP1 expression associates with tumor progression. For instance, high STIP1 expression correlates with poor overall survival in colorectal cancer patients, while STIP1 knockdown inhibits colorectal cancer cell proliferation, migration, and invasion [8]. STIP1 has also been identified as a circulating biomarker in ovarian cancer [9] and activates Wnt/β‐catenin signaling to drive hepatocellular carcinoma progression [10]. Moreover, STIP1 downregulation was shown to suppress cervical cancer cell glycolysis and proliferation [11]. Using a heat‐ stress mice model, we found heightened STIP1 expression in esophageal tissues after hot water treatment versus controls [6]. Furthermore, STIP1 is highly expressed in human ESCC tissues compared to adjacent normal tissues, and its overexpression associates with poor prognosis. Importantly, STIP1 enhances glycolytic flux in ESCC, though the specific mechanisms require further investigation.

The metabolic enzyme AHCY is highly conserved across diverse organisms including bacteria, nematodes, yeast, plants, insects, and vertebrates. AHCY functions within the ubiquitous one‐carbon metabolic cycle to enable one‐carbon transfer for biosynthesis, amino acid homeostasis, redox control, and epigenetic regulation. As S‐adenosyl‐L‐homocysteine (SAH) resembles S‐adenosyl methionine (SAM) and competitively binds methyltransferases (MTases), AHCY activity regulates MTase function through a negative feedback loop. The SAM:SAH ratio indicates cellular methylation capacity, with decreases potentially impairing methylation potential. Recent evidence suggests AHCY exhibits a key role in tumorigenesis. For instance, H19 knockdown was found to induce AHCY‐dependent changes in myotube methylation patterns at specific DNMT3B target genes [12]. Additionally, AHCY overexpression increased proliferation, clonogenicity, invasion, and angiogenesis of colon cancer cells, while its knockdown impaired these oncogenic phenotypes [13]. Furthermore, intestinal Apc deletion in mice sufficiently upregulated AHCY expression, also induced in human colorectal cancer. AHCY inhibition prevented hyperproliferation from acute Apc deletion even with mutant Kras [14]. In Nevertheless, the functional role of AHCY in esophageal cancer remains unknown, as do potential interrelationships between AHCY activity and glycolytic flux.

In this study, we demonstrate heat stimulation can alter glycolysis pathway and upregulation of STIP1 expression in esophagus tissues. STIP1 also highly expression in esophageal cancer cells and tissues and is associating with poor prognosis. STIP1 knockdown inhibited cancer cell growth in vitro and in vivo. Moreover, our study delineates a novel pathway in esophageal squamous cell carcinoma whereby STIP1 enhances the interaction between AHCY and LDHA, enabling AHCY to recruit PRMT3 to methylate LDHA at R106. This STIP1‐mediated methylation and activation of LDHA by the AHCY‐PRMT3 complex leads to heightened glycolytic flux, meeting the bioenergetic and biosynthetic demands of rapid cancer cell proliferation. In summary, STIP1 acts through AHCY in a moonlighting capacity to post‐translationally activate LDHA and reprogram cancer cell metabolism, fueling ESCC progression. Targeting this critical STIP1‐AHCY‐LDHA glycolytic axis may offer therapeutic potential in ESCC.

## 2 Materials and methods

### 2.1 Cells and Reagents

The esophageal cancer cell lines KYSE30, KYSE450, KYSE150, KYSE70, KYSE410 and KYSE510 were obtained from the Cell Bank of the Chinese Academy of Sciences (Shanghai, China). The immortalized normal esophageal epithelial cell line SHEE was generously provided by Dr. Enmin Li (Shantou University, Guangdong, China) [15, 16]. HEK293T cells were purchased from the American Type Culture Collection. All cell lines were authenticated by short tandem repeat (STR) profiling prior to use and confirmed to be mycoplasma‐free. Cells were maintained in RPMI‐1640 medium supplemented with 10% fetal bovine serum at 37°C and 5% CO_2_. LCA was purchased from MedChemExpress (Monmouth Junction, NJ).

### 2.2 Cell Proliferation Assay

To assess the effects of LCA on cell viability, esophageal cancer cells (1.5‐2 × 10^3^ per well) were seeded in 96‐well plates and exposed to a range of LCA concentrations (0‐10 µM) diluted in complete growth medium. After 24, 48 and 72 hours of incubation, cell proliferation was determined by adding MTT reagent (0.5 mg mL^−1^) to each well, incubating for 2 hours to allow MTT metabolism, then solubilizing the formazan crystals and quantifying absorbance at 570 nm as a measure of viable cells.

### 2.3 Foci Formation Assay

Cells were seeded in 6‐well plates at a density of 600 cells per well, 3 mL of growth medium was added, and incubated at 37°C for 10 days. After 10 days, colonies were stained with 0.4% crystal violet solution. Excess staining solution was rinsed off with PBS and the plates were allowed to air dry. Representative images of stained colonies were taken and the number of colonies was counted manually. The assay was performed in triplicate for each cell line.

### 2.4 Anchorage‐independent Cell Growth Assay

For anchorage‐independent cell growth assay, 8× 10^3^ esophageal cancer cells were resuspended in 0.33% agar diluted in complete growth medium containing varying LCA doses (0‐10 µM) and layered over a 0.6% agar base layer in 6‐well plates. Plates were incubated for 14 days to allow colony formation. Subsequently, colonies were observed under a wide‐field microscope and photographed. The number of colonies larger than 50 µm were counted using Image‐Pro Plus software. The assay was performed in triplicate wells for each cell line.

### 2.5 Heat Stimulation Treatment

KYSE30 and KYSE450 cells were cultured in 6 cm dishes until reaching 80% confluence, followed by medium replenishment. A metal platform was pre‐equilibrated to 42°C in a water bath. Upon temperature stabilization, the cell culture dishes were subjected to heat stimulated by placement on the pre‐warmed platform for 1 hour, followed by a 2‐hour recovery period in a 37°C incubator. Post‐recovery, cells were harvested and processed for protein extraction and subsequent Western blot analysis.

### 2.6 Surface Plasmon Resonance (SPR) Binding Assay

The binding interaction between LCA and STIP1 was analyzed by SPR using a Biacore T200 system (GE Healthcare, Waukesha, WI, USA). STIP1 protein was immobilized at a density of 200 response units on a CM5 sensor chip (#BR‐1005‐30) via amine coupling. LCA prepared in buffer containing <1% (v/v) DMSO was injected at concentrations ranging from 15.625 to 500 µM at a flow rate of 30 µL/min at 25°C. The change in refractive index due to LCA‐STIP1 binding was monitored in real‐time as response units. Sensorgrams depicting response units versus time were generated and analyzed using BIAevaluation 3.0 software to evaluate the kinetics and affinity of the LCA‐STIP1 interaction.

### 2.7 GSH and NAPDH Level Detection Assay

Collect esophageal cancer cells KYSE 30 and KYSE 450, then prepared the samples according to the instructions of the Beijing Solarbio assay kit (GSH detection kit # BC1175; NADPH detection kit #BC1100). Draw a standard curve using GSH and NADPH standards, measure the absorbance of the sample using Microplate reader at 412 nm (GSH) and 570 nm (NADPH). Calculate GSH and NADPH content based on the standard curve.

### 2.8 Cellular Thermal Shift Assay

HEK293 cells were transfected with STIP1 expression plasmids using lipofectamine 2000 and incubated for 48 hours. Cells were then treated with LCA for 4 hours, harvested into PCR tubes (100 µL) and subjected to thermal gradient incubation ranging from 45°C to 70°C (5°C increments) for 5 minutes. After two freeze‐thaw cycles in liquid nitrogen and on ice, cell supernatants were collected for subsequent western blot analysis.

**Co‐immunoprecipitation**

Cells were lysed in RIPA buffer (Solarbio) at 4°C and cell lysates were immunoprecipitated with the indicated antibodies overnight at 4°C with rotation. The following day, protein A/G agarose beads (Santa Cruz Biotechnology) were added to the lysates and incubated for 2 hours at 4°C with rotation. Beads were washed four times with RIPA buffer and immunoprecipitated complexes were eluted by boiling for 7 minutes in SDS‐PAGE loading buffer. Immunoprecipitated proteins were resolved by SDS‐PAGE and analyzed by western blot.

### 2.9 Patient‐derived Xenograft (PDX) Mouse Model

Animal studies were approved by the Ethics Committee of Zhengzhou University (Zhengzhou, Henan, China). PDX tumors were established in 6‐week‐old female NOD/SCID mice (Vital River Labs, Beijing, China) housed under pathogen‐free conditions on a 12hr light/dark cycle. Informed consent was obtained from patients to acquire fresh surgical ESCC tissues, which were implanted subcutaneously and passaged for 3 generations in mice. PDX tumor fragments (0.1‐0.12 g) were then implanted into the right flank of mice. When tumors reached approximately 100 mm^3^, mice were randomized into treatment groups. For lentiviral treatments, condensed viruses (100 µL) expressing shRNAs targeting STIP1 (shSTIP1#1/2) or scramble control were injected intratumorally every 3 days over 12 days. Vehicle (5% DMSO, 20% PEG400 in PBS) or LCA (10 or 20 mg kg^−1^) were administered via oral gavage daily. Tumor volumes were measured twice weekly. At endpoint, tumors were harvested when volumes reached approximately 1 cm^3^.

### 2.10 Cell‐derived Xenograft (CDX) Mouse Model

Female athymic nude mice aged 6–8 weeks were randomly divided into experimental groups with equal group mean weights. Cells were harvested, resuspended in cold PBS at 5 × 10^6^ cells/0.1 mL, and subcutaneously injected into the flank of each mouse using a 1 mL syringe. Tumor growth was monitored by measuring tumor dimensions using calipers and calculating tumor volume using the formula: V = 0.5 × length × width × height. Mouse weights and tumor volumes were recorded weekly. All animal procedures were performed in accordance with institutional guidelines and protocols approved by the Institutional Animal Care and Use Committee.

### 2.11 PDX‐derived Organoids (PDXO)

PDXOs were established from fresh PDX tumor fragments using methods described previously [17]. Briefly, PDX tumors were minced and digested enzymatically in HBSS with collagenase IV and Y‐27632 to obtain a single‐cell suspension. Cells were filtered, trypsinized, strained through a 70 µm filter, washed in basal medium, and viable cells quantified by Trypan blue exclusion. To initiate PDXO culture, 2 × 10^4^ viable dissociated cells were seeded per well in 24‐well plates in Matrigel matrix and maintained in organoid medium. Medium was refreshed every 2–3 days and organoid growth monitored microscopically. Once PDXOs reached approximately 30 µm diameter on day 14 of culture, lipofectamine‐mediated transfection was performed with non‐targeting control shRNA or an shRNA pool targeting STIP1. Organoids were incubated with MTT reagent. Absorbance was measured to quantify relative cell proliferation compared to untreated controls.

### 2.12 4NQO‐induced Esophageal Cancer in Wild‐type and *Stip1*‐conditional Knockout Mice

*Stip1*‐conditional knockout mice were purchased from Cyagen Biosciences Inc (Suzhou, China). Mice were genotyped by PCR analysis with specific primers (Supplementary Table 1) according to the protocol of Cyagen Biosciences. Wild‐type and *Stip1*‐cKO mice were randomly allocated to the control (water) or treatment (4NQO) group. Mice in the treatment group received 4NQO (Sigma, Cat#N8141) in their drinking water at a concentration of 100 mg/L. The 4NQO‐containing drinking water was replaced weekly, and the mice were weighed during this process. After 16 weeks of 4NQO treatment, the mice were switched to regular sterile water until they were euthanized at 28 weeks. Upon euthanasia, the esophageal tissue was harvested and divided into two parts: one part for histological examination (H&E staining) and the other for immunohistochemical (IHC) analysis.

### 2.13 Western Blotting (WB) Experiment

The cells and tissues were lysed using RIPA buffer on ice for 2 hours. Protein concentrations were determined by the BCA assay (Solarbio, Beijing, China). The WB operation method was consistent with our previous procedure [18]. The antibodies we used were provided in supplementary Table 2. Protein bands were visualized using a chemiluminescence (ECL) detection reagent (GE Healthcare Life Science, Little Chalfont, HP, UK).

### 2.14 Immunohistochemistry (IHC) Assay

Formalin‐fixed paraffin‐embedded tumor tissues were sectioned and processed for IHC staining using a rabbit detection kit (Cat#SP9001, ZSGB‐BIO). Antigen retrieval and blocking were performed according to the manufacturer's protocol. Tissue sections were incubated with primary antibodies against STIP1, AHCY, and Ki67 (50 µL per section) overnight at 4°C. Subsequently, sections were washed, incubated with horseradish peroxidase‐conjugated secondary antibodies for 30 minutes at room temperature, and developed using 3,3’‐diaminobenzidine (DAB) substrate. Sections were counterstained with hematoxylin, then imaged and quantified using Image Pro Plus 6.0 and Aperio ImageScope software for density of positive staining.

### 2.15 Immunofluorescence Staining

Cells grown on glass coverslips were fixed with 4% paraformaldehyde, permeabilized with 0.2% Triton X‐100, and blocked with goat serum. Cells were then incubated overnight at 4°C with primary antibodies targeting STIP1 (Proteintech, 68155‐1‐Ig, 1:200 dilution) and AHCY (Proteintech, 10757‐2‐AP, 1:100 dilution). Subsequently, cells were incubated with fluorophore‐conjugated secondary antibodies including Alexa Fluor 488‐labeled goat anti‐mouse IgG (Invitrogen, A11029, 1:200 dilution) to detect STIP1 and Alexa Fluor 568‐labeled donkey anti‐rabbit IgG (Invitrogen, A10042, 1:200 dilution) to detect AHCY. Nuclei were counterstained with DAPI (Solarbio). Coverslips were mounted onto slides and imaged by confocal microscopy (Nikon) to assess co‐localization of STIP1 and AHCY expression.

### 2.16 Protein Expression and Purification

For recombinant protein purification, 3×Flag‐tagged constructs were expressed in Escherichia coli BL21 cells. Bacteria were induced with 0.5 mM IPTG at 16°C overnight to express the recombinant proteins. Cells were lysed by sonication in lysis buffer (50 mM NaH_2_PO_4_, 300 mM NaCl, 20 mM imidazole, 1 mM PMSF, 1% Triton X‐100). Lysates were incubated with Ni‐NTA agarose resin (QIAGEN) for 4 hours at 4°C to bind Flag‐tagged proteins. Resin was washed four times with wash buffer (50 mM NaH_2_PO_4_, 300 mM NaCl, 60 mM imidazole). Purified proteins were then eluted using elution buffer containing higher imidazole concentration (50 mM NaH_2_PO_4_, 300 mM NaCl, 300 mM imidazole). Purity of eluted proteins was verified by SDS‐PAGE followed by Coomassie blue staining.

### 2.17 Pull Down Assay and Mass‐spectrometry

For pull‐down assays using KYSE450 and KYSE510 lysates, STIP1 protein complexes were immunoprecipitated with anti‐STIP1 antibody or normal IgG control and isolated using Protein A/G beads. Precipitated proteins were separated by SDS‐PAGE, and entire gel lanes were excised and subjected to in‐gel trypsin digestion overnight at 37°C with shaking. Resulting peptides were extracted and analyzed by liquid chromatography‐tandem mass spectrometry (LC‐MS/MS) to identify STIP1‐interacting proteins [19, 20]. For pull‐down assays assessing LCA binding, DMSO or LCA were conjugated to 4B‐sepharose beads and incubated with equal amounts of cancer cell lysates overnight at 4°C with rotation. Precipitated STIP1 levels were assessed by western blotting.

### 2.18 Computer Docking Model

Molecular modeling and docking studies were performed using Schrödinger Suite 2015 software. The crystal structure of STIP1 was obtained from the RCSB Protein Data Bank (PDB) and prepared using the Protein Preparation Wizard in Schrödinger using default parameters. The structure of LCA was prepared for docking using LigPrep [21]. Molecular docking of LCA to the STIP1 binding site was performed with Glide using standard precision mode. The best docking poses were visualized using Maestro.

### 2.19 Sam/ Sah Ratio Calculation

KYSE410 cells were harvested and washed with phosphate‐buffered saline (PBS) two times. Subsequently, the cell lysate underwent centrifugation at 1000 rpm for 3 minutes to facilitate separation and removal of the supernatant fraction. Quantification of SAM and SAH levels was performed utilizing the SAM and SAH ELISA Combo Kit in accordance with the manufacturer's recommended protocol. The SAM/SAH ratio was then calculated to assess the methylation potential.

### 2.20 Tests of Glucose Uptake, LDH Activity, and Lactate Export

To assess glycolytic flux, esophageal cancer cells were seeded in 6‐well plates and cultured for 24 hours at 37°C in a humidified 5% CO_2_ incubator. Following treatments, culture media was collected and cells were lysed. Glucose uptake, lactate secretion, and LDH activity were measured using colorimetric assay kits according to manufacturer's protocols (Glucose assay kit, ab235976, Abcam; LDH assay kit, A020‐2‐2, Jianchengbio; Lactic acid assay kit, A019‐2‐1, Jianchengbio). In brief, glucose levels were determined based on glucose oxidase‐mediated conversion of glucose to gluconic acid and hydrogen peroxide, which reacts with a chromogenic substrate. Lactate secretion was measured by lactate oxidase‐driven reaction of lactate with a probe to generate a product detectable at 530 nm. LDH activity was determined by following the LDH‐catalyzed reduction of NAD^+^ to NADH, which converts a tetrazolium dye to a reduced form measurable at 450 nm. Absorbance values were normalized to total cellular protein quantified using a BCA protein assay kit. All measurements were performed in triplicate.

### 2.21 Oxygen Consumption and ECAR

Extracellular acidification rates (ECAR) of ESCC cells were analyzed using a Seahorse XF24 Extracellular Flux Analyzer (Agilent). Cells were seeded at 30,000 cells/well on a 24‐well Seahorse XF24 plate and allowed to adhere overnight. Prior to analysis, growth medium was replaced with assay medium. Using pre‐loaded cartridges, glucose (10 mM), oligomycin (1 µM), and 2‐deoxyglucose (2‐DG, 50 mM) were sequentially injected into wells at specified timepoints. ECAR measurements were recorded throughout and data was analyzed using Seahorse XF24 software. ECAR measures lactic acid secretion reflecting glycolytic flux.

### 2.22 In Vivo AHCY Activity Assay

AHCY enzyme activity was measured in a 96‐well format using a commercial assay kit (HBP37963, Huabangbio, Shanghai), according to the manufacturer's protocol. This assay quantifies AHCY activity through detection of homocysteine levels in cell lysates. Briefly, KYSE450 cells were washed with cold PBS and lysed directly in plates with 200 µL lysis buffer (40 mM hexadecyltrimethylammonium bromide, 75 mM Tris‐HCl pH 8.0, 1 M NaCl, 15 mM EDTA). Lysates were cleared by centrifugation at 15,000 × g for 15 minutes at 4°C. Immediately after, 100 µL supernatant was used to determine AHCY activity by measuring homocysteine concentration colorimetrically as a surrogate for AHCY‐mediated hydrolysis of S‐adenosylhomocysteine into adenosine and homocysteine. Absorbance was read using a FilterMax F3&F5 Multi‐Mode Microplate Reader (Molecular Devices).

### 2.23 Real‐time PCR

Total RNA was extracted using TRIzol reagent per the manufacturer's protocol and reverse transcribed into cDNA using the PrimeScript RT Reagent Kit (Cat#RR047A, Takara). AHCY mRNA expression was quantified by real‐time quantitative PCR (qPCR) using the SYBR Premix Ex Taq II kit (Cat#RR420A, Takara) on a CFX96 Touch Real‐Time PCR system (BioRad). The following primers were used to amplify human *AHCY*: forward 5’‐ATCCTCAAGGTGCCTGCCATCA‐3’, reverse 5’‐CGGCAATCATCACATCTGTGGC‐3’. AHCY mRNA levels were normalized to *GAPDH* housekeeping reference gene. Relative expression was calculated using the 2^−ΔΔCt^ method.

### 2.24 Quantification and Statistical Analysis

Quantitative data are presented as mean ± standard deviation from at least 3 independent experiments. Statistical comparisons between two groups were performed using an unpaired two‐tailed Student's t‐test. One‐way or two‐way analysis of variance (ANOVA) followed by appropriate post‐hoc tests were used for comparisons involving multiple groups and/or conditions. Overall survival analysis was conducted using Kaplan‐Meier curves and significance between subgroups was determined by the log‐rank test. P values less than 0.05 were considered statistically significant.

## 3 Results

### 3.1 Heat‐stimulation Promotes Glycolysis and Induces STIP1 Overexpression

**FIGURE 1:**
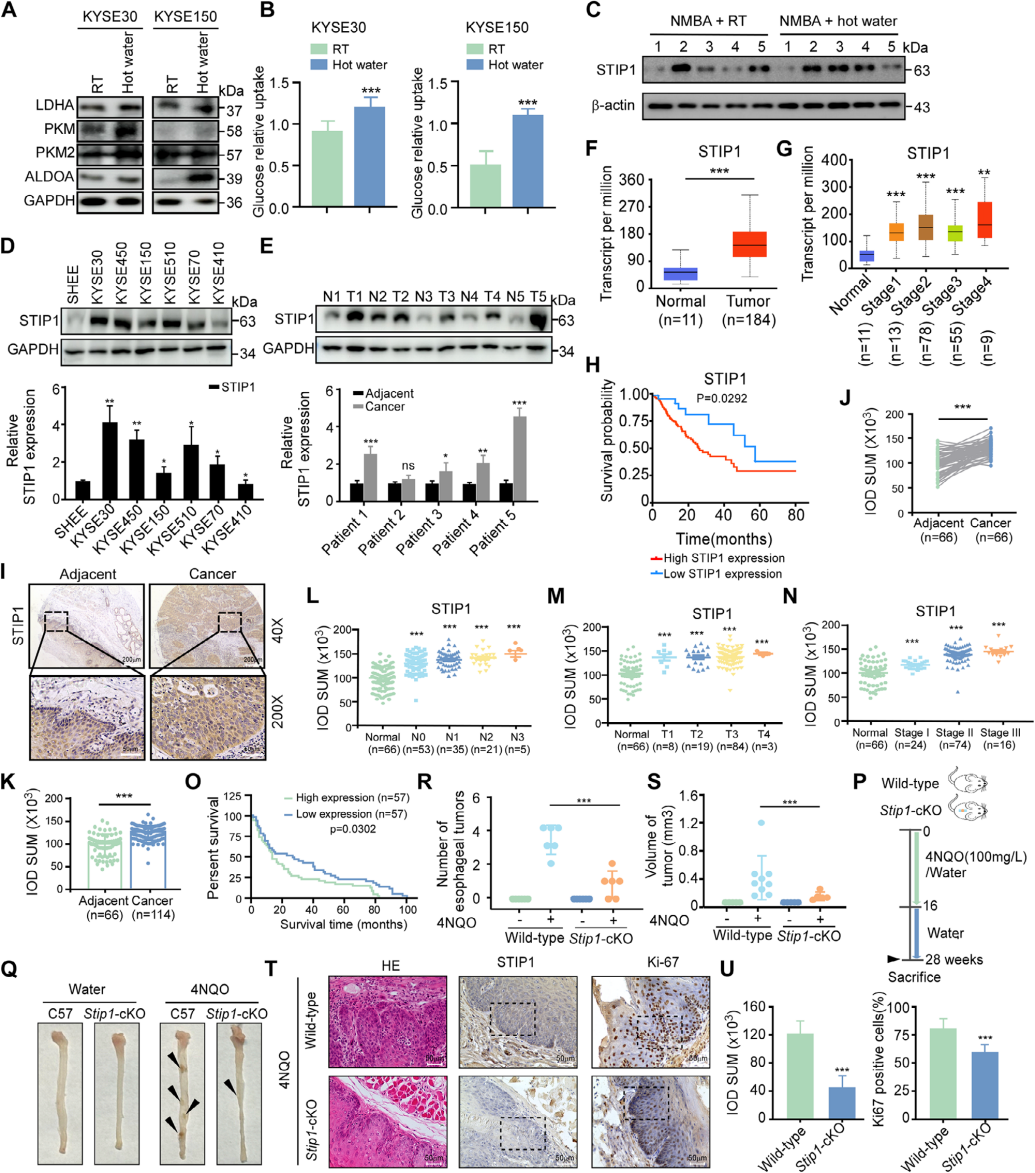
Heat‐stimulation promotes glycolysis and induces STIP1 overexpression. A. WB experiment was performed to measure the glycolysis enzymes level. **B**. Glucose uptake was detected by kit after 65°C water stimulation. **C**. STIP1 expression was heightened in esophageal tissues of mice treated with hot water compared to normal controls. **D**. Across a panel of human esophageal cell lines, STIP1 protein levels were elevated in multiple ESCC lines versus normal esophageal epithelial cells (upper panel). The bands intensities were subjected to quantitative analysis (lower panel). **E**. In analysis of 5 paired patient ESCC tumor and adjacent normal tissues, STIP1 protein was increased in tumor samples compared to matched normal epithelium (upper panel). The bands intensities were subjected to quantitative analysis (lower panel). TCGA database was used to evaluate STIP1 expression across normal and cancerous tissues (**F**), examining its distribution across tumor stages (**G**) and investigating correlations between STIP1 expression and patient prognostic outcomes (**H**). **I**. By immunohistochemistry of ESCC tissue microarrays, STIP1 staining was more intense in tumor areas versus paired normal epithelium (40x and 100x magnifications shown). Quantitative analysis confirmed significantly higher STIP1 protein levels in ESCC tumors compared to paired normal tissues(**J**) and in unpaired ESCC versus normal esophagus (**K**). Stratifying by clinicopathologic characteristics, STIP1 expression was incrementally increased with higher lymph node involvement (**L**), poorer tumor grade (**M**), and advanced tumor stage (**N**). **O**. Relationship between STIP1 expression level and overall survival for tissue microarray. **P**. Schematic of the 4NQO‐induced esophageal cancer model. **Q**. Representative images of esophageal tissues harvested from WT and STIP1 cKO mice treated with 4NQO/water. Arrows indicate carcinomas. **R,S**. Quantification of tumor numbers and volume in WT versus STIP1 cKO mice after 4NQO treatment. One‐way ANOVA, mean ± SD, *p < 0.01, **p < 0.001.**T**. IHC staining analysis of STIP1 and Ki67 expression in esophageal tissues (Scale: 50 µm). **U**. Quantified protein expression levels, unpaired t‐test, mean ± SD, ***p < 0.001. Statistical analysis was performed using Student's paired t test in J. Student's unpaired t test in K, L, M and N. Data represent mean ± SD.

While we previously reported that heat stimulation is a key factor in esophageal cancer carcinogenesis, the underlying mechanism remains poorly understood. In order verify the mechanism, we analysis glycolysis pathways in ESCC cells after heat stimulation. Results showed that glucose uptake was increased and glycolysis pathway was active after heat stimulation (Figure 1A, B). To further delineate the molecular mechanisms underlying heat stimulation, we performed RNA sequencing analysis on heat‐exposed mouse esophageal tissues from our previously established model [6]. Among the differentially expressed genes, STIP1 emerged as the sole upregulated gene significantly associated with stress response pathways. Given previous reports linking STIP1 to glycolytic regulation [11], this finding positioned STIP1 as a compelling candidate mediator of heat‐induced metabolic alterations (Figure S1A). Subsequently, we validated STIP1 expression in heat‐treated esophageal tissues, confirming our RNA‐Seq findings of significant STIP1 upregulation following heat stimulation (Figure 1C). Further investigation revealed elevated STIP1 expression across multiple ESCC cell lines compared to normal esophageal epithelial cells (Figure 1D). Western blot analysis of 5 paired patient ESCC tumor and adjacent normal tissue samples, collected from patients during surgery at the Affiliated Cancer Hospital of Zhengzhou University, further demonstrated increased STIP1 protein expression in the tumor samples (Figure 1E). To comprehensively investigate STIP1's oncogenic potential, we analyzed the TCGA database. The results demonstrated that STIP1 was highly expressed in ESCC tissues compared to normal tissues, maintained high expression across different tumor stages, and its elevated expression was significantly associated with poor patient prognosis (Figure 1F–H). To further confirm the role of STIP1 in ESCC, we used a commercial ESCC tissue microarray containing 66 paired ESCC tissues and adjacent tissues for IHC. Immunohistochemical staining of ESCC tissue microarrays revealed higher STIP1 expression in tumor versus paired normal esophageal tissues (Figure 1I–J). STIP1 was also elevated in unpaired ESCC tumors compared to normal tissues (Figure 1K). Stratifying by clinicopathologic features, STIP1 was upregulated in ESCCs with greater lymph node involvement (Figure 1L), larger tumor size (Figure 1M), and more advanced clinical stage (Figure 1N) (Table 1). Moreover, high STIP1 expression correlated with poor overall survival in ESCC patients (Figure 1O). To further investigate the function of STIP1 in vivo, we generated a conditional *Stip1‐*knockout (*Stip1*‐cKO) and WT mice model and induced esophageal tumor formation through treatment with the carcinogen 4‐nitroquinoline 1‐oxide (4NQO) (Figure 1P). After 28 weeks of 4NQO exposure, esophageal tissues were harvested and quantified tumor incidence. The cancerous lesions which were induced by 4NQO were observed in the esophagus (Figure 1Q, black arrow). Compared to WT mice, the *Stip1*‐cKO group exhibited a significant reduction in the number of ESCC tumors (Figure 1R) and tumor size (Figure 1S) upon 4NQO treatment. Furthermore, we conducted hematoxylin and eosin (H&E) staining and IHC analysis to evaluate the expression levels of Ki67, a well‐established proliferation marker. Compared with WT mice group, STIP1 and Ki‐67 were reduced in *Stip1*‐cKO mice (Figure 1T–U). Collectively, these results demonstrate that heat‐stimulation promote glycolysis and induced STIP1 abnormal expression. And our results highlight an oncogenic role for STIP1 in promoting ESCC tumorigenesis.

**TABLE 1:** Correlation between STIP1 expression and clinicopathologic characteristics of ESCC.

Characteristics	STIP1 expression level		*P*
	Low(*n* = 57)	High(*n* = 57)	
Gender			0.671
Male	41 (35.96%)	43 (37.72%)	
Female	16 (14.04%)	14 (12.28%)	
Age			0.453
≥65	32 (28.07%)	28 (24.56%)	
<65	25 (21.93%)	29 (25.44%)	
Clinical stage			<0.001*
I	24 (21.05%)	2 (1.75%)	
II	24 (21.05%)	45 (39.47%)	
III	9 (7.89%)	10 (8.77%)	
N classification			0.252
N0	31 (27.19%)	22 (19.30%)	
N1	13 (11.40%)	22 (19.30%)	
N2	11 (9.65%)	10 (8.77%)	
N3	2 (1.75%)	3 (2.63%)	
T classification			0.365
T1	5 (4.63%)	4 (3.51%)	
T2	10 (5.56%)	9 (7.89%)	
T3	42 (39.81%)	41 (35.97%)	
T4	0 (0.00%)	3 (2.63%)	

### 3.2 STIP1 promotes Cell Growth and PDXO Development

**FIGURE 2:**
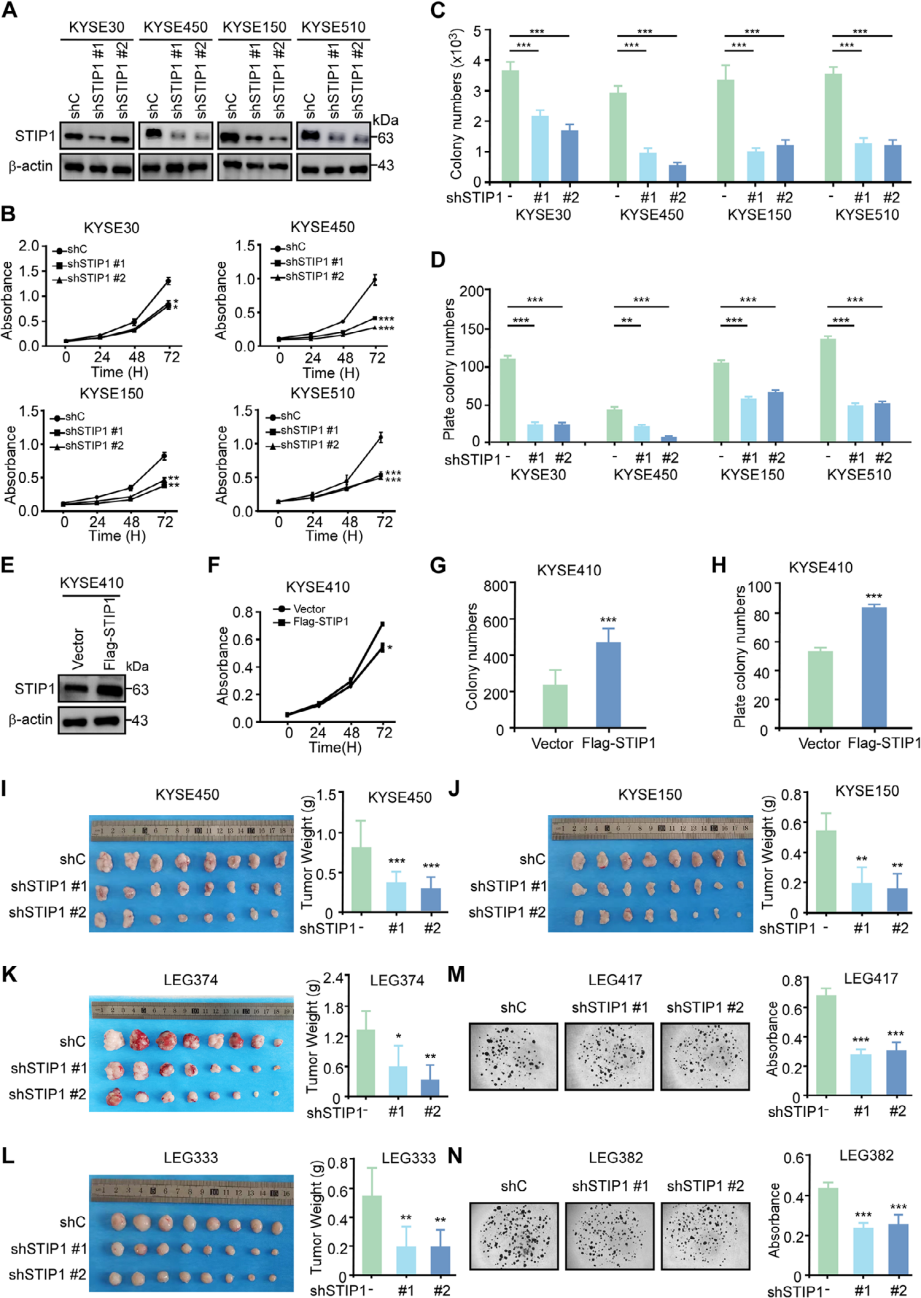
STIP1 promotes cell growth and PDXO development. **A**. Stable STIP1 knockdown ESCC cell lines were generated, with efficient STIP1 protein reduction confirmed by western blotting. **B**. STIP1 knockdown significantly decreased ESCC cell proliferation based on MTT assays. **C**. **D** Anchorage independent growth and plate colony formation assay from different cells with STIP1 knockdown. Colonies were counted using Image Pro‐Plus (Scale bar: 200 µm). **E**. STIP1 was overexpressed in KYSE410 cells and the expression of STIP1 was determined by western blot. **F**. After overexpressed STIP1, cell proliferation was measured by MTT assay. **G**, **H**. Anchorage independent growth and plate colony formation assay from KYSE410 with STIP1 overexpression. Colonies were counted using Image J‐Plus (Scale bar: 200 µm). **I**‐**J** Esophageal squamous cell carcinoma cells with stable knockdown of STIP1 expression via short hairpin RNAs (shRNAs) or non‐targeting control shRNA were subcutaneously injected into athymic nude mice (n = 8 per group) to evaluate effects on tumorigenicity. Tumor weights were calculated in the right panel. **K**‐**L**. Intertumoral injection of STIP1 shRNA in PDXs suppressed tumor growth compared to control shRNA. Tumor weights were calculated in the right panel. **M‐N**. STIP1 knockdown reduced the number and size of PDXOs. Organoids absorbance was measured in the right panel. All data statistical differences were evaluated using Student's t‐test. *p < 0.05, **p < 0.01, ***p < 0.001.

To investigate the functional role of STIP1 in ESCC, we stably knocked down STIP1 expression using shRNAs in KYSE30, KYSE450, KYSE150, and KYSE510 cell lines, which exhibit high endogenous STIP1 protein levels. Western blot analysis verified that the protein expression level was significantly decreased in ESCC cells after knock‐ down of STIP1 (Figure 2A). STIP1 knockdown significantly decreased cell proliferation in these lines as measured by MTT assay (Figure 2B). Anchorage‐dependent growth was also reduced following STIP1 knockdown, as shown by decreased colony formation in clonogenic assays (Figure 2C; Figure S2A) and foci formation assays (Figure 2D; Figure S2B). Conversely, STIP1 overexpression in KYSE410 cells, which have relatively low basal STIP1 protein levels, increased cell proliferation and clonogenicity (Figure 2E–H; Figure S2C‐D). The rescue experiment demonstrated that overexpression of STIP1 could alleviate the inhibition of cell proliferation caused by STIP1 knockdown (Figure S2E). To further investigate the tumorigenic potential of STIP1 in ESCC cells, we employed a CDX model in which STIP1‐silenced cells were subcutaneously inoculated into immunocompromised mice. The results demonstrated that STIP1 knockdown significantly attenuated tumor growth in the ESCC CDX models. The tumor weight and volume in the shSTIP1‐inoculated cohort were markedly reduced compared to the shC‐inoculated control group (Figure 2I–J; Figure S2F, I), while the average body weight of mice remained comparable across groups (Figure S2G, J). Immunohistochemical analysis of the CDX tumors revealed diminished Ki67 positivity, indicative of decreased proliferation, upon STIP1 knockdown (Figure S2H, K). Additionally, we validated the functional role of STIP1 in ESCC progression using a PDX model. The clinical information of PDXs were shown in Figure S2L. Following shSTIP1 virus inoculation, the final average tumor weight in the shSTIP1 groups was reduced relative to the shC group (Figure 2K, L). Moreover, the tumor size in the shSTIP1 groups was considerably smaller than that in the shC group (Figure S2M, P), while the mice body weights remained unaltered (Figure S2N, Q). Consistent with the ESCC tumor burden, Ki‐67 expression was also significantly decreased in shSTIP1 groups compared to that in shC group (Figure S2O, R). Moreover, STIP1 knockdown reduced the proliferation of PDXOs (Figure 2M, N). Together, these in vitro and in vivo findings demonstrate that STIP1 plays an oncogenic role in promoting ESCC cell proliferation and tumor growth.

### 3.3 STIP1 plays a Crucial Role in Glycolysis

**FIGURE 3:**
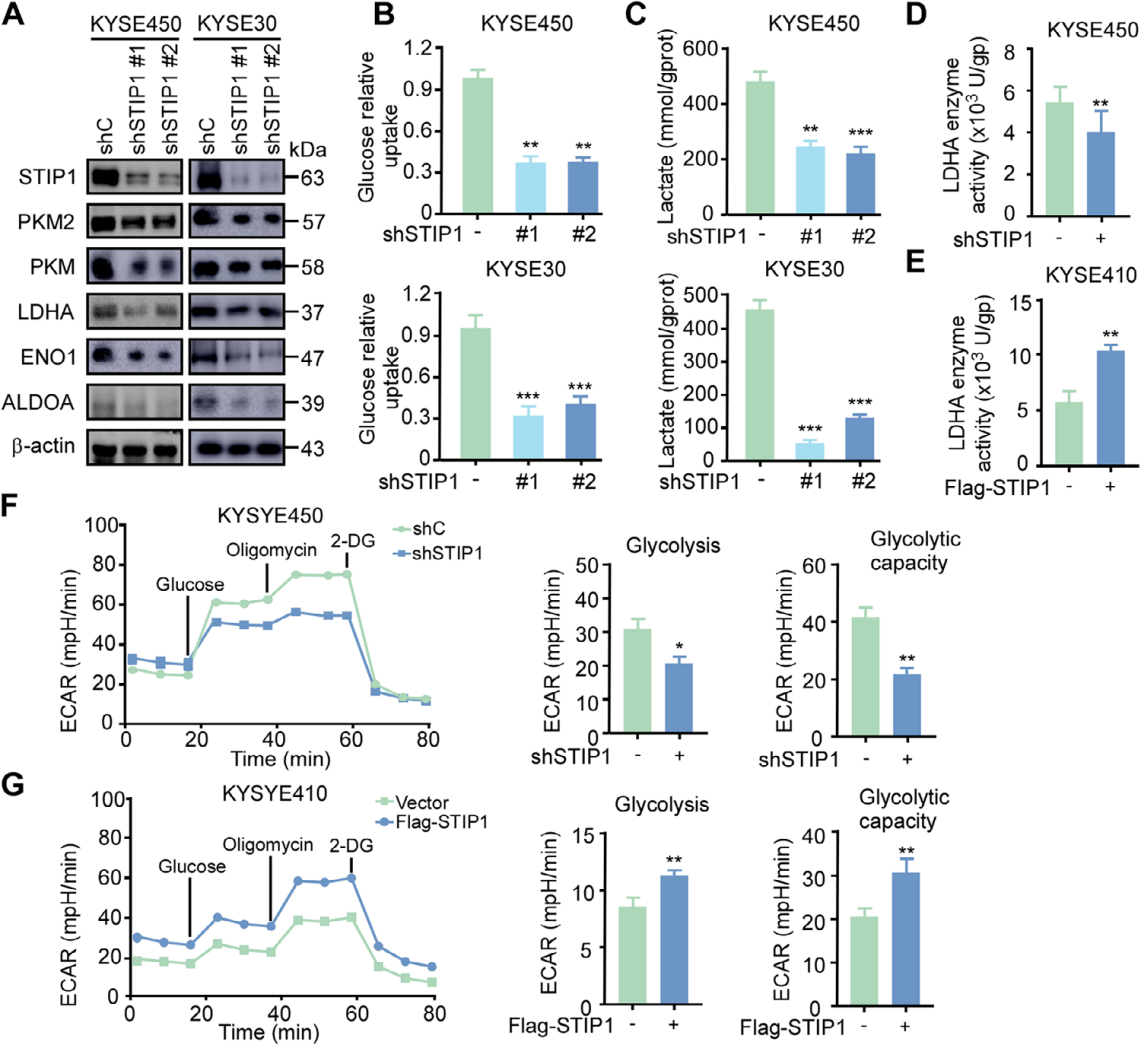
STIP1 plays a crucial role in glycolysis. **A**. Western blot shows STIP1 knockdown in KYSE450 and KYSE30 cells reduce protein levels of key glycolytic enzymes including PKM2, PKM, LDHA, ENO1 and ALDOA in ESCC cells. β‐actin is shown as a loading control. **B**. STIP1 knockdown decreases glucose consumption in ESCC cells. Values are normalized to total protein content. **C**. STIP1 knockdown reduces lactate secretion in ESCC cells. Lactate levels in conditioned media were normalized to cell number. **D**. STIP1 knockdown lowers LDHA catalytic activity in ESCC cell lysates. **E**. Overexpression STIP1 enhance LDHA enzyme activity. LDHA activity was determined by measuring NADH levels spectrophotometrically. **F,G**. ECAR reflecting glycolytic flux, is decreased or increased after STIP1 knockdown in KYSE450 or overexpression in KYSE410 cells. ECAR was measured using a Seahorse XFe96 analyzer under basal conditions and in response to glucose, oligomycin, and 2‐DG. Data are mean ± SD of triplicate experiments. *p < 0.05, **p < 0.01, ***p < 0.001 by Student's t‐test.

Metabolic reprogramming is a hallmark of cancer, with most tumor cells becoming highly glycolytic for energy production and to support rapid proliferation [22, 23, 24]. Firstly, we demonstrated that heat‐stimulation promote glycolysis and induced STIP1 overexpression, but the function of STIP1 in glycolysis need to further verify. To explore whether STIP1 regulates metabolic reprograming, we found that STIP1 knockdown in KYSE450 and KYSE30 ESCC cells reduced protein levels of glycolytic enzymes including PKM2, LDHA, ENO1 and ALDOA (Figure 3A). Functionally, STIP1 knockdown inhibited glucose uptake and lactate secretion, suggesting impaired glycolysis (Figure 3B, C). As LDHA catalyzes the final step of glycolysis by converting pyruvate to lactate and has known oncogenic functions in promoting the Warburg effect [25, 26, 27], we assessed LDHA catalytic activity. STIP1 knockdown decreased LDHA enzyme activity in ESCC cells (Figure 3D), while overexpression of STIP1 enhanced LDHA enzyme activity (Figure 3E). Using Seahorse extracellular flux analysis, STIP1 knockdown cells exhibited reduced ECAR (Figure 3F). These effects on ECAR were rescued by STIP1 re‐expression (Figure 3G). Collectively, through modulation of glycolytic enzyme expression and activity, our results demonstrate that STIP1 is a key driver of the Warburg effect and glycolytic metabolism in ESCC cells.

### 3.4 STIP1 interacts with AHCY to Enhance Binding to LDHA, Thereby Promoting Glycolytic Flux

**FIGURE 4:**
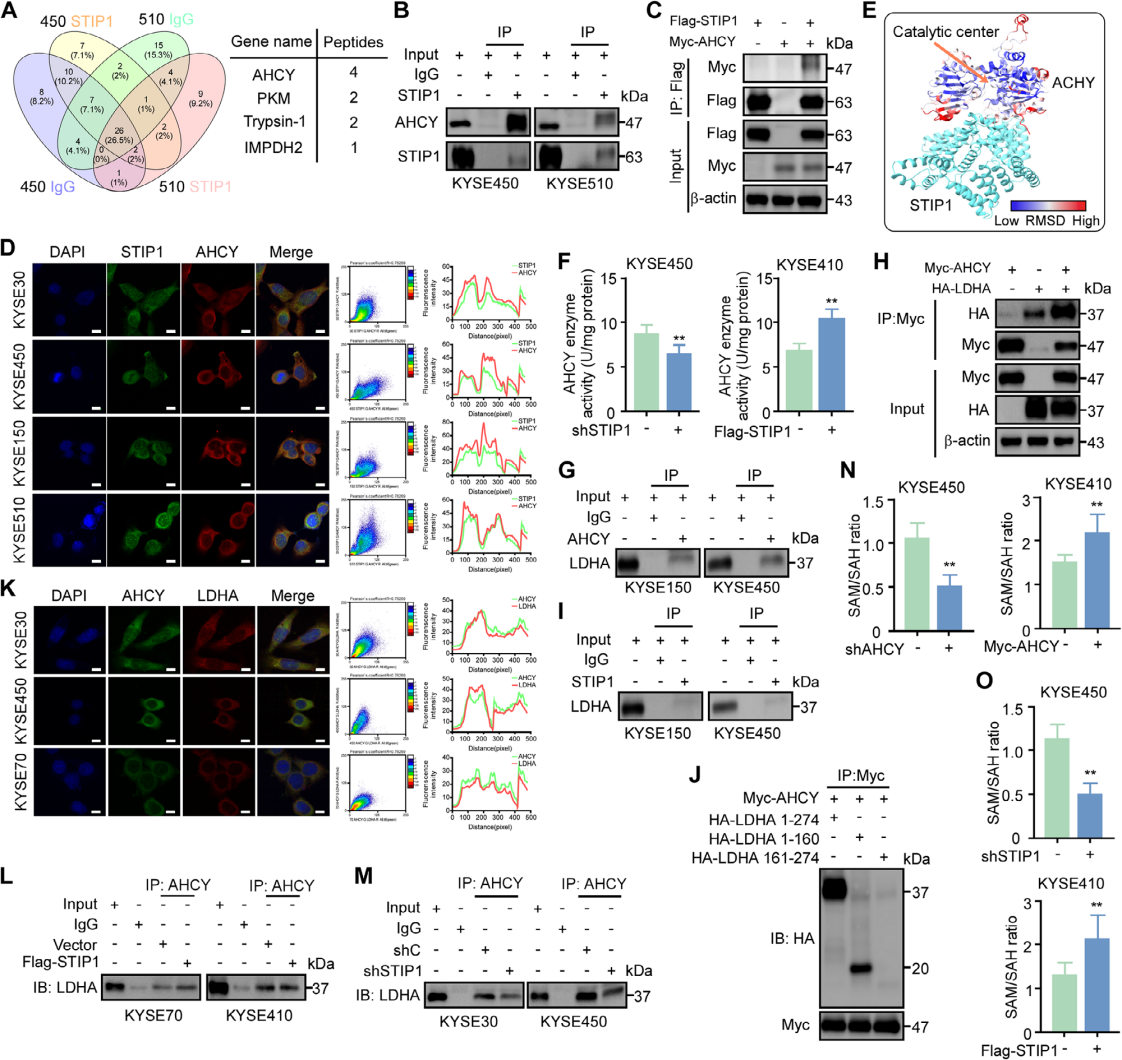
STIP1 interacts with AHCY to enhance binding to LDHA, thereby promoting glycolytic flux. **A**. Mass spectrometry analysis identifies glycolytic enzymes as STIP1‐interacting proteins. **B**, **C**. Endogenous and exogenous co‐immunoprecipitation verifies binding between STIP1 and AHCY **D**. Immunofluorescence staining shows co‐localization of STIP1 (green) and AHCY (red) in the cytoplasm of ESCC cells (DAPI stain in blue). **E**. The AHCY‐STIP1 complex was modeled using Alphafold3. Then the AHCY in the model was aligned to the experimental structure (PDB 1LI4), and colored by C‐alpha RMSD using ChimeraX. Red indicates high RMSD and larger conformational change. **F**. Knockdown or overexpression of STIP1 reduces or enhances AHCY enzyme activity, respectively. **G,H**. Co‐immunoprecipitation confirms endogenous and exogenous interaction between AHCY and LDHA. **I**. Endogenous IP assay to check the binding of STIP1 and LDHA. **J**. HEK293T cells were transfected with Myc‐tagged AHCY and truncated LDHA including 1–274, 1–160, 161–274. Immunoprecipitation was performed using anti‐HA affinity gel followed by western blots using anti‐Myc antibody. **K**. Immunofluorescence shows cytoplasmic co‐localization of AHCY (green) and LDHA (red) in ESCC cells. **L,M**. STIP1 knockdown or overexpression reduces or enhances the AHCY‐LDHA interaction. **N,O**. Knockdown or overexpression of STIP1 or AHCY decreases or increases the SAM:SAH ratio. Data are mean ± SD of 3 experiments. *p < 0.05, **p < 0.01, ***p < 0.001 by Student's t‐test.

To investigate the mechanism of STIP1 in esophageal cancer progression, we performed pull‐down coupled with mass spectrometry (MS) analysis. Following the Venn diagram analysis, we prioritized AHCY, PKM, Trypsin‐1 and IMPDH2 as candidate genes based on the peptides score (Figure 4A). Endogenous and exogenous co‐immunoprecipitation confirmed direct binding between STIP1 and AHCY (Figure 4B–C). Pearson correlation analysis and fluorescence intensity correlation analysis on immunofluorescence (IF) images to demonstrate the co‐localization of STIP1 and AHCY in the cytoplasm (Figure 4D). While STIP1 knockdown did not alter AHCY protein or mRNA expression (Figure S3A‐B). IHC analysis to evaluate the expression levels of ALDOA, ENO1, LDHA and PKM2 compared with WT mice group. These glycolysis markers were reduced in Stip1‐cKO mice (Figure S3C). We hypothesized that STIP1 binding may induce conformational changes in AHCY to modulate its enzyme activity. In order to verify this hypothesis, an AlphaFold‐based modeling tool was used, conformational changes in AHCY upon binding to STIP1 were predicted to occur near the contact site with STIP1 and in the vicinity of the catalytic center (Figure 4E). Using a commercial AHCY activity assay, we found that STIP1 knockdown dramatically reduced AHCY catalytic activity and overexpression STIP1 enhanced AHCY catalytic activity (Figure 4F). Together, these results demonstrate that STIP1 directly interacts with and activates AHCY enzyme function in ESCC cells through conformational modulation rather than altered AHCY expression.

We found that AHCY, whose activity is enhanced by STIP1 binding. we hypothesized that AHCY‐interacting proteins involved in glycolysis enzymes to regulate glycolysis process. Endogenous and exogenous IP assay showed that AHCY interacts with the glycolytic enzyme LDHA, but not with other key glycolytic enzymes (Figure 4G, H, Figure S3D). In contrast, our findings indicated that STIP1 does not bind to LDHA (Figure 4I). To identify the critical region in LDHA responsible for its interaction with AHCY, we conducted co‐immunoprecipitation experiments using HEK‐293T cells overexpressing Myc‐tagged AHCY and various truncated versions of HA‐tagged LDHA. Our findings revealed that the N‐terminal region of LDHA, spanning amino acid residues 1 to 160 (LDHA 1–160), is essential for its binding to AHCY (Figure 4J). Pearson correlation analysis and fluorescence intensity correlation analysis revealed that AHCY and LDHA co‐localization in the cytoplasm (Figure 4K). Notably, Overexpression of STIP1 increased the interaction between AHCY and LDHA, whereas STIP1 knockdown diminished the AHCY‐LDHA interaction. (Figure 4L–M). Collectively, these findings indicate that STIP1 facilitates the interaction between AHCY and LDHA.

As AHCY catalyzes the hydrolysis of SAH, a byproduct and inhibitor of methyltransferases, it regulates transmethylation reactions and the SAM/SAH ratio indicating cellular methylation capacity [28]. We found STIP1 or AHCY knockdown reduced the SAM/SAH ratio, while their overexpression increased it (Figure 4N, O). In summary, our results demonstrate STIP1 binds and activates AHCY, stimulating its interaction with LDHA to promote glycolysis. STIP1‐AHCY constitute an axis regulating the SAM/SAH ratio and methylation capacity in ESCC cells.

### 3.5 AHCY Recruited PRMT3 to Methylate LDHA at R106 to Enhance LDHA Enzyme Activity

**FIGURE 5:**
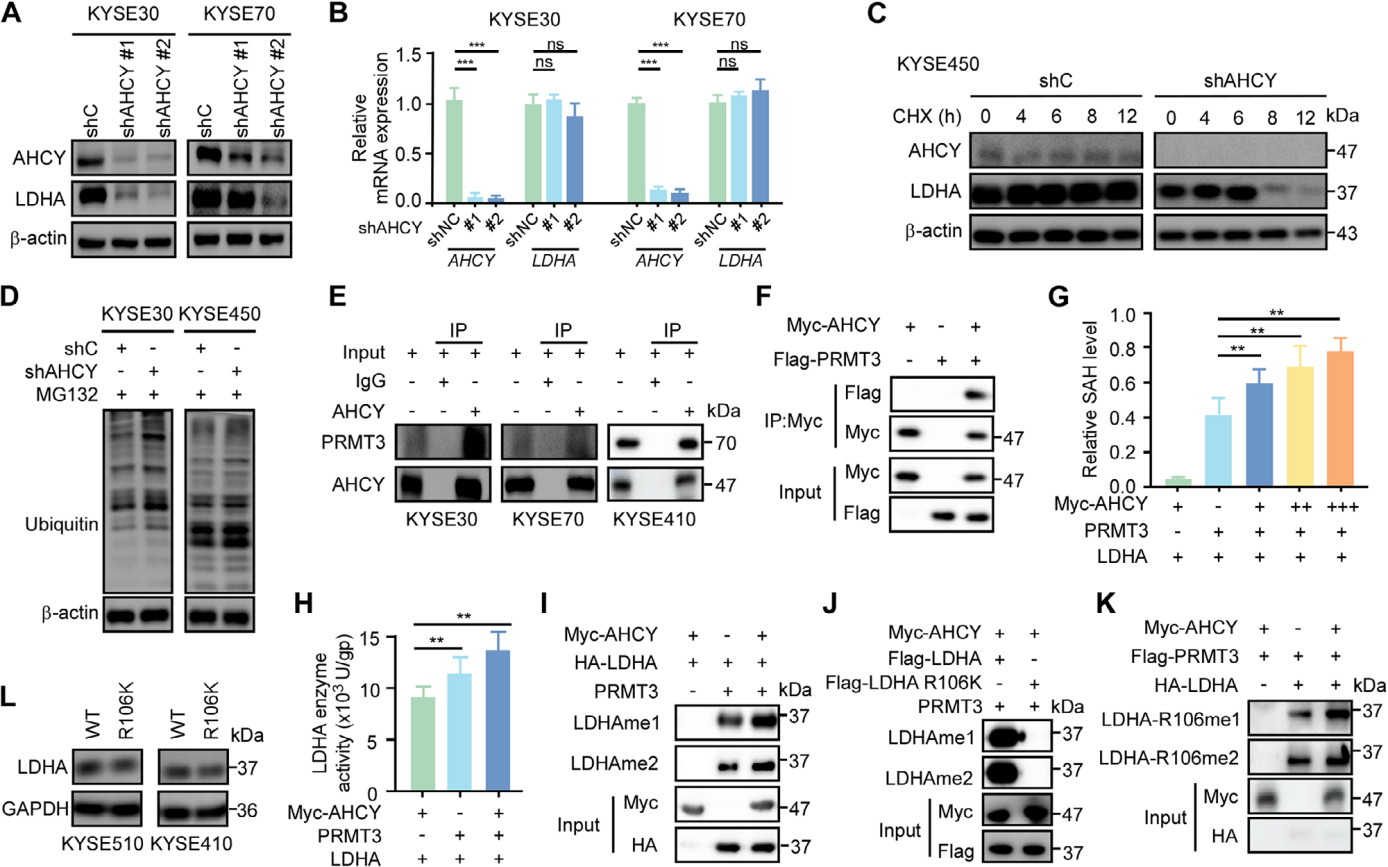
AHCY recruited PRMT3 to methylate LDHA at R106 to enhance LDHA enzyme activity. **A**. Western blot shows knockdown AHCY decrease LDHA protein levels in ESCC cells, respectively. β‐actin is shown as a loading control. **B**. qPCR analysis to check mRNA level of AHCY and LDHA after AHCY knockdown. GAPDH was used as reference gene. **C**. AHCY knockdown accelerates LDHA protein degradation in presence of CHX. LDHA levels were determined by western blotting at indicated timepoints after CHX treatment. **D**. Proteasome inhibitor MG132 rescues LDHA protein levels upon AHCY knockdown in KYSE450 and KYSE30 cells. **E,F**. Co‐immunoprecipitation verifies endogenous and exogenous interaction between AHCY and PRMT3 in ESCC cells. **G**. AHCY enhances PRMT3 enzyme activity in a dose‐dependent manner, measured by quantifying reaction product SAH. **H**. AHCY presence increases LDHA catalytic activity, determined by measuring NADH levels spectrophotometrically. **I**. In vitro methylation assay shows AHCY recruits PRMT3 to methylate LDHA. Methylation was detected by immunoblotting using pan‐monomethylation and pan‐asymmetric dimethylation antibody. **J**. Mutating LDHA R106 to lysine prevents methylation by AHCY‐recruited PRMT3. **K**. R106K mutation abrogates LDHA methylation at R106me1 and R106me2, as determined using site‐specific methylation antibodies. **L**. R106K mutant LDHA exhibits reduced protein levels by western blot.

We next investigated the functional consequences of AHCY‐LDHA interaction. AHCY knockdown decreased LDHA protein levels without affecting LDHA mRNA expression (Figure 5A, B), indicating post‐transcriptional regulation. AHCY depletion accelerated LDHA degradation in the presence of cycloheximide (CHX), suggesting reduced protein stability (Figure 5C). Treatment with the proteasome inhibitor MG132 rescued LDHA levels upon AHCY knockdown (Figure 5D), indicating involvement of proteasomal degradation. Together, these results demonstrate that AHCY binding protects LDHA from ubiquitin‐proteasome mediated degradation, thereby regulating its protein stability in ESCC cells.

It was previously reported that protein arginine methyltransferase 3 (PRMT3) binds and methylates LDHA [29]. Here, we find AHCY interacts with PRMT3 by co‐immunoprecipitation (Figure 5E‐F). Functionally, AHCY binding stimulated PRMT3 enzymatic activity (Figure 5G) and LDHA catalytic function (Figure 5H). In vitro methylation assays demonstrate AHCY recruits PRMT3 to catalyze LDHA monomethylation and asymmetric dimethylation at arginine residues (Figure 5I). Using a combination of bioinformatic prediction tools (http://msp.biocuckoo.org/userguide.php) and in vitro methylation assays confirmed that mutating LDHA R106 to kinetically unfavorable lysine (R106K) abolished LDHA methylation at R106me1 and R106me2 catalyzed by the AHCY‐PRMT3 complex, results showed that R106 as the critical LDHA methylation site targeted by the AHCY‐PRMT3 complex (Figure 5J). To specifically detect LDHA methylation at R106, we generated monoclonal antibodies against R106 monomethylation (R106me1) and asymmetric dimethylation (R106me2) (Shanghai LabEx Biotechnology Co.). Using these site‐specific antibodies, in vitro methylation assays confirmed that AHCY recruit PRMT3 to methylated LDHA at R106 (Figure 5K). Compared to wildtype LDHA, R106K mutant LDHA protein levels were reduced (Figure 5L), and R106K‐expressing ESCC cells showed decreased proliferation and clonogenicity (Figure S4A‐C). Taken together, these mechanistic data delineate a pathway wherein AHCY recruits PRMT3 to methylate LDHA at R106, enhancing its stability and glycolytic function to promote ESCC growth.

### 3.6 AHCY Upregulation Promotes ESCC Progression by Enhancing LDHA Enzyme Activity

AHCY hydrolyzes S‐adenosylhomocysteine to adenosine and L‐homocysteine, playing a key role in regulating cellular methylation capacity. To explore the functional significance of AHCY in ESCC, we first examined its expression across ESCC cell lines. Compared to normal esophageal epithelial cells, AHCY was upregulated in multiple ESCC lines (Figure S5A). Analysis of 4 paired patient tumor and adjacent normal esophageal tissues, which were collected from patients during surgery at the Affiliated Cancer Hospital of Zhengzhou University, results showed heightened AHCY expression in ESCC tumors (Figure S5B). Analysis of public databases (http://ualcan.path.uab.edu/) revealed AHCY is transcriptionally upregulated across several cancer types, including ESCC, compared to normal tissues (Figure S5C). We also used the TCGA database to analyze the AHCY protein expression levels. Results showed that AHCY was highly expressed in ESCC tissues, increased across different tumor stages, and high AHCY expression was associated with poor prognosis (Figure S5D‐F). By immunohistochemistry of ESCC patient tissue microarrays, we confirmed heightened AHCY protein expression in tumors versus adjacent normal epithelium (Figure S5G). Quantitative analysis of staining intensity showed significantly higher integrated optical density (IOD) of AHCY immunostaining in cancer tissues (Figure S5H). Stratifying by tumor stage, we found the highest AHCY expression in Stage II and III ESCCs compared to earlier stages (Figure S5I). These data showed that AHCY highly expressed in ESCC and correlated with poor prognosis. Subsequently, we utilized the TCGA database to evaluate LDHA expression levels. Our analysis revealed that LDHA highly expressed in ESCC tissues (Figure S5J), showed a progressive increase in expression levels across advancing tumor stages (Figure S5K), and demonstrated a substantial correlation between high LDHA expression and diminished patient survival outcomes (Figure S5L). Because STIP1 elevated expression was also significantly associated with poor patient prognosis (Figure. 1F–H), we performed correlation analysis on STIP1, AHCY and LDHA, and the analysis results show that there is a correlation between STIP1 and AHCY and LDHA (Figure S5M‐O).

**FIGURE 6:**
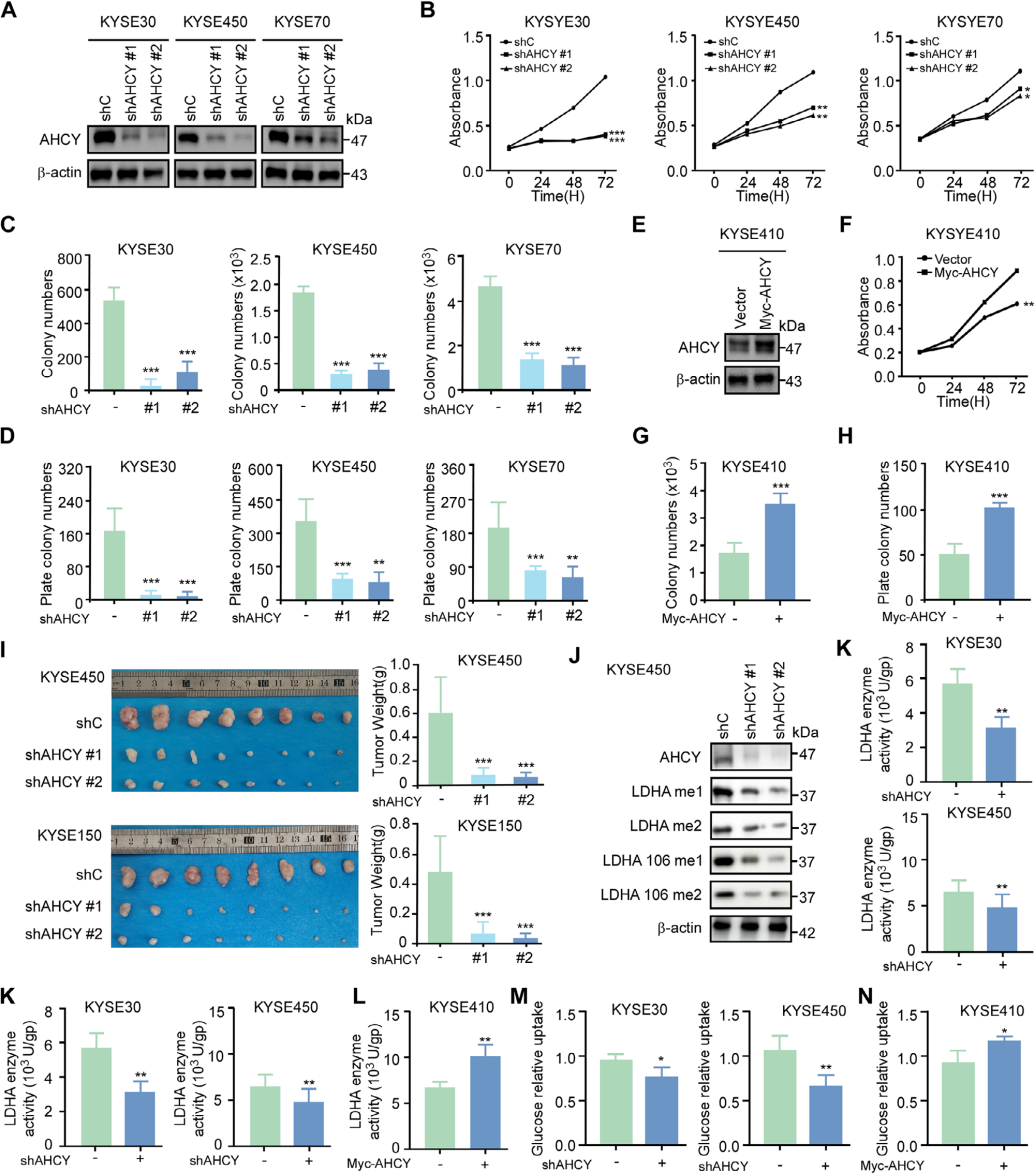
AHCY upregulation promotes ESCC progression by enhancing LDHA stability and glycolytic activity. **A**. Western blot confirms efficient AHCY knockdown using two independent shRNAs in ESCC cell lines. **B**. AHCY knockdown reduces ESCC cell proliferation by MTT assay. **C**. Clonogenic assay shows AHCY knockdown decreases anchorage‐independent growth of ESCC cells. **D**. AHCY knockdown reduces foci formation of ESCC cells. **E**. Western blot validates AHCY overexpression in KYSE410 cells after lentiviral transduction. **F–H**. AHCY overexpression increases ESCC cell proliferation, clonogenicity, and foci formation. **I**. In cell line‐derived xenografts, AHCY knockdown cells form smaller tumors with reduced weights compared to control cells. Tumor weight was shown in right panel. **J**. AHCY knockdown reduces LDHA methylation at R106me1 and R106me2 marks. **K,L**. AHCY modulates LDHA enzyme activity in ESCC cells. **M,N**. Glucose consumption is altered by AHCY knockdown and overexpression. Data are mean ± SD of 3 experiments. *p < 0.05, **p < 0.01, ***p < 0.001 by Student's t‐test.

To directly assess the functional role of AHCY in ESCC, we used three independent shRNAs to stably knock down its expression in ESCC cell lines, which was verified by western blotting (Figure 6A). AHCY silencing dramatically reduced ESCC cell proliferation and clonogenicity (Figure 6B–D; Figure S6A, B). Conversely, AHCY overexpression in KYSE410 cells increased cell growth and colony formation (Figure 6E‐H; Figure S6C–D). In CDX models, AHCY knockdown markedly suppressed ESCC tumor growth without affecting body weight (Figure 6I; Figure S6E, F). IHC analysis revealed that silencing AHCY expression led to a significant decrease in the levels of Ki‐67, a well‐established marker of cellular proliferation (Figure S6G, H). All these results demonstrated that AHCY play an oncogenic role in ESCC.

Mechanistically, knockdown of AHCY led to a reduction in LDHA methylation level (Figure 6J), knockdown of AHCY also led to a reduction in LDHA enzyme activity (Figure 6K), whereas its overexpression enhanced LDHA enzyme activity (Figure 6L). Furthermore, AHCY knockdown led to a reduction in glucose uptake in KYSE30 and KYSE450 cells (Figure 6M), whereas its overexpression enhanced glucose uptake in KYSE410 cells (Figure 6N).

Collectively, these functional data demonstrate that aberrant AHCY upregulation promotes ESCC progression by enhancing LDHA enzyme activity and glycolytic activity. Targeting this novel AHCY‐LDHA signaling axis may offer therapeutic potential.

### 3.7 LCA Is a STIP1 Inhibitor

**FIGURE 7:**
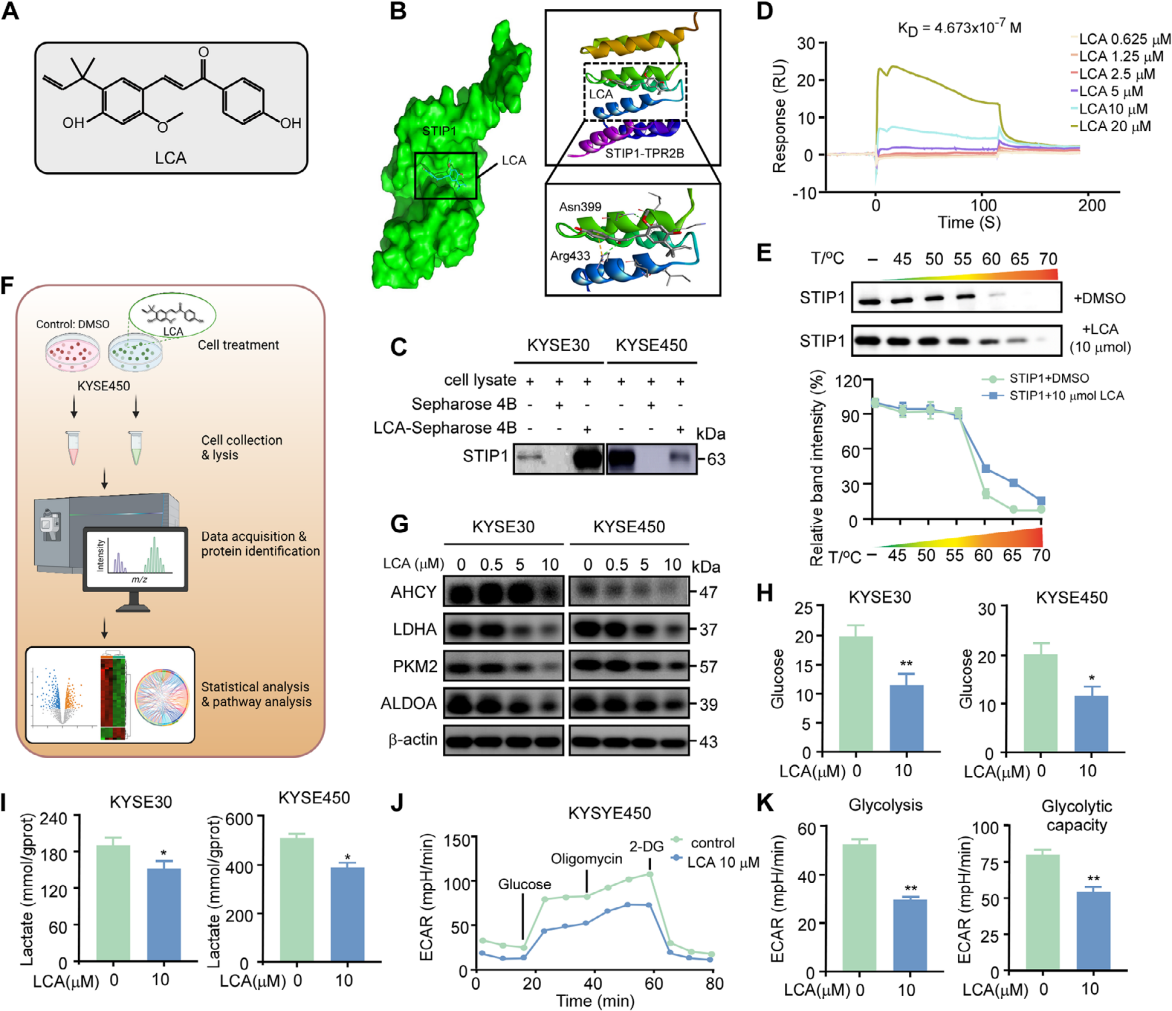
LCA is a STIP1 inhibitor. **A**. Chemical structure of LCA. **B**. Computational modeling predicts binding between LCA and STIP1. **C**. Pull‐down assay shows LCA directly interacts with STIP1 protein in vivo. **D**. SPR analysis verifies direct binding between LCA and STIP1. Sensorgram shows response over time as LCA passes over STIP1‐conjugated chip. **E**. Cellular thermal shift assay demonstrates LCA binds and stabilizes STIP1 protein in ESCC cells. **F**. Overview of quantitative proteomics approach. KYSE450 cells were treated with LCA or DMSO control for 24 h, followed by tandem mass tag (TMT) labeling and LC‐MS/MS analysis. **G**. After treat various doses of LCA, AHCY, LDHA, PKM2 and ALDOA protein level were measured by western blot. **H,I**. LCA decreases glucose consumption and LDH vitality in ESCC cells. **J,K**. ECAR is lowered by LCA, reflecting impaired glycolysis. Data are mean ± SD of 3 experiments. *p < 0.05, **p < 0.01, by Student's t‐test.

As STIP1 exhibits oncogenic functions in ESCC tumorigenesis and glycolysis, we performed in silico screening of a natural compound library to identify potential STIP1 inhibitors. This computational molecular docking approach identified LCA (Structure in Figure 7A) as a candidate STIP1‐targeting compound. Molecular modeling corroborated favorable binding between LCA and STIP1 (Figure 7B). We verified this interaction using in vitro pull‐down assays with KYSE30 and KYSE450 cells (Figure 7C). SPR analysis further demonstrated direct LCA‐STIP1 binding (Figure 7D). Additionally, cellular thermal shift assays showed LCA stabilization of STIP1 protein, consistent with direct binding (Figure 7E). Together, these biophysical approaches validate LCA as a natural compound that directly interacts with STIP1 protein.

To assess LCA‐induced proteomic changes that may result from STIP1 binding, we performed quantitative proteomics on KYSE450 cells treated with LCA versus DMSO control for 24 hours (Figure 7F). We identified 349 differentially expressed proteins using a ≥2‐fold change cutoff, including 277 upregulated and 72 downregulated proteins upon LCA treatment (Figure S7A‐C). Pathway enrichment analysis of these LCA‐regulated proteins revealed effects on diverse metabolic processes, prominently including glycolysis (Figure S7D). The broad impact on metabolic signaling pathways is consistent with LCA disrupting STIP1‐mediated glycolytic regulation in ESCC cells. These unbiased proteomic data provide additional evidence that LCA alters cancer cell metabolism through direct binding to STIP1.

We next assessed the effects of LCA on glycolytic regulation in ESCC cells. Treatment of KYSE30 and KYSE450 cells with increasing LCA doses reduced protein levels of glycolytic enzymes including LDHA, PKM2, and ALDOA, also decrease the expression level of AHCY (Figure 7G). Functionally, LCA decreased glucose consumption, lactate secretion, and ECAR reflective of glycolytic flux (Figure 7H‐K). LCA also decreased STIP1 protein expression, without affecting STIP1 mRNA levels based on RT‐PCR analysis (Figure S7E, F). Using the protein synthesis inhibitor cycloheximide (CHX), we found LCA shortened the half‐life of STIP1 protein compared to vehicle control (Figure S7G, H). Moreover, the proteasome inhibitor MG132 attenuated LCA‐mediated STIP1 degradation (Figure S7I).

Together, these results demonstrate LCA reduces expression of glycolytic proteins and glycolytic activity in ESCC cells. Mechanistically, LCA decreases STIP1 protein stability through stimulation of proteasome‐mediated degradation.

### 3.8 LCA Inhibits ESCC Progression in Vitro and in Vivo

**FIGURE 8:**
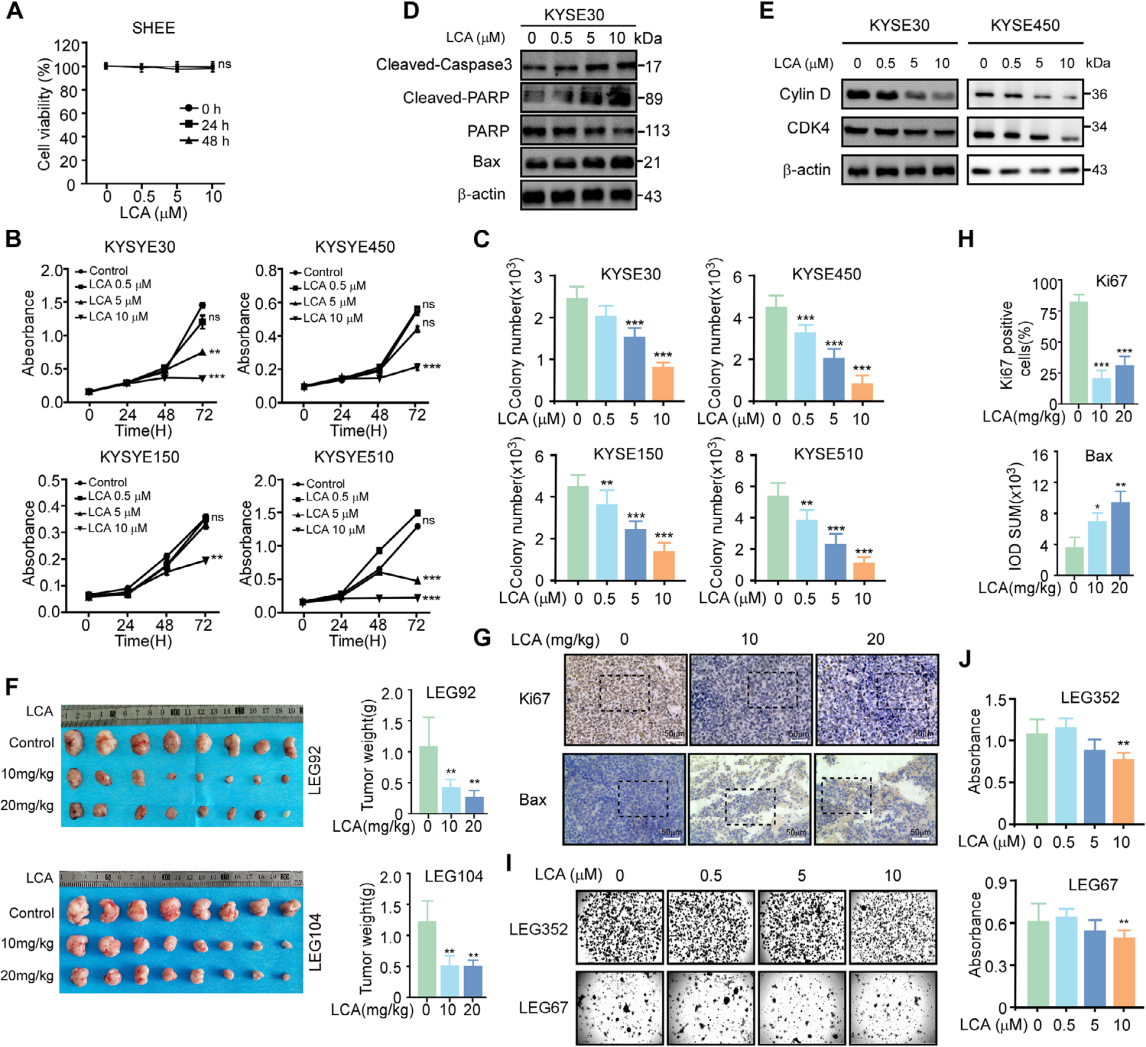
LCA inhibits ESCC progression in vitro and in vivo. **A**. MTT assay shows no cytotoxicity of LCA against normal esophageal epithelial SHEE cells. **B**. LCA dose‐dependently reduces ESCC cell proliferation by MTT assay. **C**. Soft agar assay demonstrates LCA inhibits anchorage‐independent growth and colony formation of ESCC cells. Quantified colony numbers. **D**. Western blot analysis shows LCA induces apoptosis markers including cleaved‐PARP and cleaved‐caspase‐3, and upregulates pro‐apoptotic Bax. **E**. cell cycle markers were detected by western blot. **F**. The tumor tissues obtained from patients were subcutaneously injected into SCID mice, and then mice were given with LCA (10 mg kg^−1^, 20 mg kg^−1^) or vehicle four times per week. The mice were sacrificed and tumors mass were isolated from mice. Tumor weight was shown in right panel. **G,H**. IHC staining of PDX tumors shows LCA decreases proliferation marker Ki67 and increases apoptosis marker Bax. Quantification of IHC staining intensity is shown. **I,J**. In PDXO models, LCA suppresses organoid growth and viability in a dose‐dependent manner. Data are mean ± SD of 3 experiments. *p < 0.05, **p < 0.01, ***p < 0.001 by Student's t‐test.

We first assessed the effects of LCA on normal esophageal epithelial cells, which showed no cytotoxicity (Figure 8A). In ESCC cell lines (KYSE30, KYSE450, KYSE150 and KYSE510), LCA dose‐dependently reduced cell proliferation by MTT assay (Figure 8B) and clonogenicity in soft agar and focus formation assays (Figure 8C; Figure S8A–C). LCA also induced apoptosis and G1 cell cycle arrest with dose‐dependent reduction in cyclin D1and CDK4 (Figure 8D‐E; Figure S8D–G).

To further validate the effects of LCA in vivo, PDX models were employed, and the clinical information for the PDX models LEG92 and LEG104 is presented in Figure S9A. Results showed that LCA significantly decreased tumor volumes and weights in two ESCC PDX models without effect the mice body weight (Figure 8F; Figure S9B‐E). Immunohistochemistry of LCA‐treated PDX tumors showed reduced proliferation marker Ki67 and increased apoptosis marker cleaved‐caspase 3 (Figure 8G‐H). Lastly, LCA dose‐dependently inhibited growth of PDX‐derived organoids, further demonstrating its anti‐tumor efficacy (Figure 8I‐J).

Collectively, these in vitro and in vivo data demonstrate that STIP1 promote the binding between AHCY and LDHA, then AHCY recruit PRMT3 to methylated LDHA at R106 to promote glycolysis to promote ESCC progression. The anti‐neoplastic effects of STIP1 inhibitor LCA against ESCC models through inhibition of cell proliferation, induction of apoptosis, and suppression of tumor growth (Figure 8I).

## 4 Discussion

Metabolic reprogramming is a hallmark of cancer, with enhanced glycolysis playing a critical role in promoting cancer progression [30]. One of the best characterized metabolic alterations in cancer cells is the Warburg effect, whereby cancer cells become heavily reliant on aerobic glycolysis to meet their bioenergetic and biosynthetic demands, despite adequate oxygen availability. This metabolic switch to enhanced glycolytic flux plays a critical role in enabling the rapid proliferation of cancer cells by providing not only ATP but also glycolytic intermediates that support synthesis of nucleotides, amino acids, lipids and other macromolecules. Targeting deregulated cancer cell metabolism, such as glucose uptake and glycolysis, has therefore emerged as a promising therapeutic strategy [31, 32, 33]. However, deeper understanding of the molecular drivers underlying metabolic reprogramming in specific cancer contexts is still needed. In this paper, we found heat‐stimulation promote glycolysis and induced STIP1 overexpression. STIP1 knockdown inhibited glucose consumption, LDHA activity and ECAR, reflecting impaired glycolysis and mitochondrial respiration. STIP1 knockdown or overexpression inhibited or promoted ESCC cell proliferation and tumor growth in vivo. STIP1 knockdown also suppressed growth of patient‐derived organoids. Together, these data demonstrate STIP1 is a key driver of the Warburg effect in ESCC and serves as a prognostic biomarker.

AHCY catalyzes hydrolysis of SAH to sustain transmethylation reactions by removing inhibitory feedback [34].AHCY deficiency causes a rare multisystem disorder underscoring its importance in metabolism [35, 36]. AHCY inhibition was reported to reduce cancer cell proliferation [37]. AHCY also associates with chromatin and promotes circadian transcriptional programs through timely SAH hydrolysis [38]. However, the functional role of AHCY in ESCC is unknown. Here, we demonstrate that AHCY is overexpressed in ESCC, enhances LDHA stability and glycolytic activity, and its inhibition suppresses ESCC proliferation and tumor growth. Our results highlight AHCY as a promising therapeutic target in ESCC.

LDHA catalyzes the final step of glycolysis, converting pyruvate to lactate, and is frequently overexpressed across diverse cancer types including breast, lung, prostate, pancreatic, brain and liver cancers [24, 25, 26, 39, 40]. LDHA overexpression plays a critical role in promoting cancer progression, and its inhibition suppresses tumor growth. LDHA can be upregulated transcriptionally by oncoproteins like HIF‐1α and c‐Myc, as well as through post‐transcriptional and post‐translational mechanisms [24]. Clinically, high LDHA expression correlates with increased metastasis and poor outcomes in cancer patients, highlighting its potential as a prognostic biomarker and therapeutic target. As a key glycolytic enzyme, the oncogenic functions of LDHA have been attributed to its role in promoting aerobic glycolysis and the Warburg effect. While prior studies have focused on transcriptional and translational control of LDHA, post‐translational regulation by modifications such as methylation remain less explored in cancer. Our study identifies a novel mechanism of LDHA post‐translational activation through methylation at R106 by AHCY‐recruited PRMT3, revealing a previously unknown layer of glycolytic regulation that promotes esophageal squamous cell carcinoma progression.

LCA is a natural chalcone compound derived from the root of Glycyrrhiza inflata. Prior studies have shown LCA exhibits anti‐cancer effects in various contexts, including suppressing PD‐L1 in lung cancer [41] inhibiting HIF‐1α and respiration in colon cancer [42]. and targeting STAT3 signaling in ovarian cancer [43]. However, the potential therapeutic efficacy and mechanisms of LCA in ESCC have been unclear. In this study, we demonstrate for the first time that LCA inhibits ESCC cell proliferation, clonogenicity, and tumor growth in patient‐derived xenografts. Mechanistically, LCA binds to STIP1 and stimulates its degradation, thereby inhibiting the STIP1‐AHCY‐LDHA glycolytic axis elucidated in our study. Our results suggest LCA holds promise as a natural anti‐tumor compound with efficacy against ESCC progression and metabolism. Further preclinical development and evaluation of LCA as part of combination regimens for ESCC treatment are warranted.

## 5 Conclusion

Our study elucidates a previously unrecognized oncogenic signaling axis in esophageal squamous cell carcinoma whereby the co‐chaperone STIP1 and metabolic enzyme AHCY are co‐opted to drive upregulation of the Warburg effect and promote ESCC progression. We demonstrate STIP1 is overexpressed in ESCCs where it enhances aerobic glycolysis. Mechanistically, STIP1 activates AHCY enzymatic function and stimulates the AHCY‐LDHA interaction, leading to LDHA stabilization and hyperactivation. This metabolic reprogramming fuels the biosynthetic demands of rapid cancer cell proliferation. The discovery of this pathway sheds light on the molecular underpinnings of metabolic deregulation in ESCC and suggests the STIP1‐AHCY‐LDHA axis may serve as a prognostic biomarker and present a novel therapeutic target opportunity. Moreover, we identified the natural compound LCA as a first‐in‐class inhibitor of oncogenic STIP1 signaling. Further development of LCA or similar small molecules to target aberrant metabolism driven by STIP1 and AHCY overexpression may offer new therapeutic strategies against ESCC. Our study provides a rationale for continued investigation into the clinical utility of therapeutically disrupting the STIP1‐AHCY glycolytic pathway in ESCC patients.

## Author Contributions

G.G.J, Y.M.S and M.Y.Y conceived and designed the experiments; G.G.J,Y.M.S and M.Y.Y assisted with designing the figures and writing; S.B.F, Y.S, K.X.Z, M.L, Q.X.G, X.Y.J and Z.W.W helped to inoculate the PDX mice and breed knockout mice; C.J.Z provided samples and tissue microarrays; K.D.L, X.L and S.M.Z edited the manuscript, M.H.L polished the manuscript; Z.P.G and Z.G.D edited the manuscript, supervised the studies and allocated the funding.

## Conflicts of Interest

The authors declare no conflicts of interest.

## Ethics Approval and Consent to Participate

All animal experiments and clinical samples in this project were conducted in accordance with the Helsini Declaration and were approved by the Ethics Committee of the China‐US (Henan) Hormel Cancer Institute (Zhengzhou, Henan, China) (CUHCI2021046) and all cancer tissues used in the study were from cancer patients.

## Supporting information



Supporting Information

Supporting Information

## Data Availability

Data sets supporting the conclusions of this article are included in this article and its accompanying files.
