# Supplementary material for: STIP1 drives Metabolic Reprogramming in Esophageal Squamous Cell Carcinoma via AHCY‐LDHA Axis
Source: Exploration (Beijing). 2025 May 25;5(5):20240198. doi: 10.1002/EXP.20240198 (PMC12561186; doi:10.1002/EXP.20240198)
Supplement: Supplementary file 1 — Supporting Information [file EXP2-5-20240198-s002.docx]

**
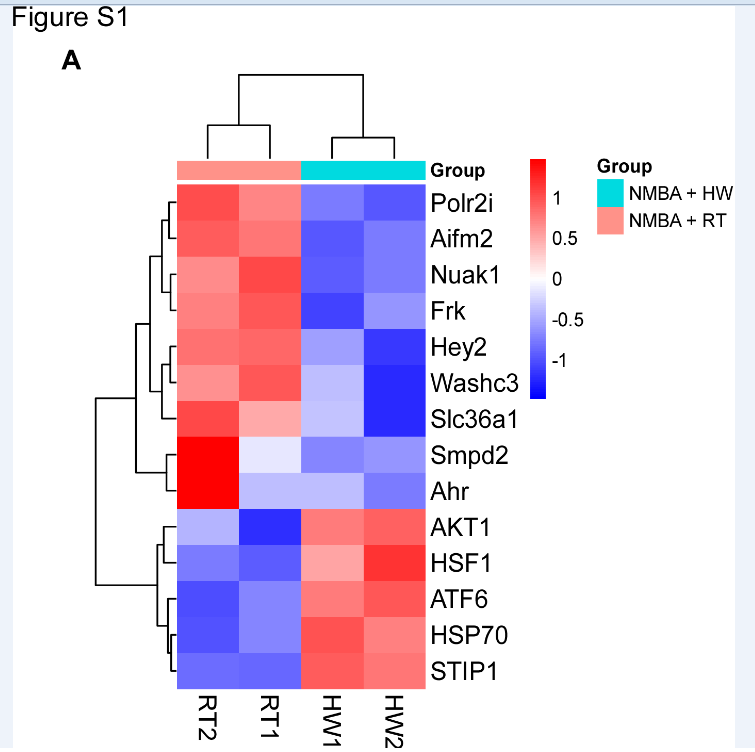
**

**Supplemental Figure 1. The heat stimulation treatment assay sequence analysis**

**A**. The heat stimulation treatment assay sequence analysis was performed in heat-stimulated esophageal tissues compared to controls in a mouse model.

**
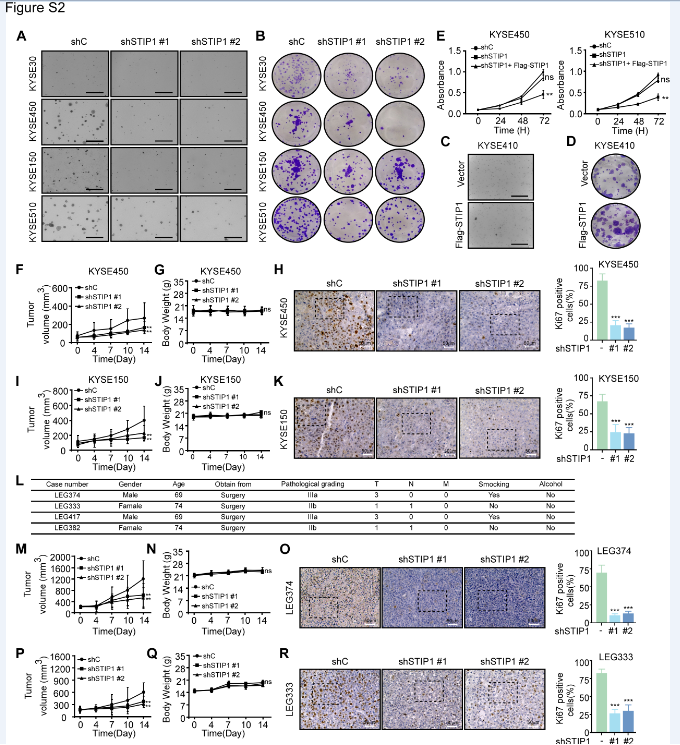
**

**Supplemental Figure 2. STIP1 knockdown inhibits ESCC tumor growth *in*** ***vitro* and *in vivo***

**A-D.** Soft agar assay and plate colony formation assay to assess anchorage-independent growth after STIP1 knockdown or overexpression. **E.** MTT assay for assessing the functionality of STIP1 in ESCC cells. **F-J**. In CDXs, ESCC cells expressing control shRNA or STIP1 shRNAs were injected subcutaneously into athymic nude mice (n=8/group). Tumor volumes were measured twice weekly using calipers. Mouse body weights were also monitored throughout the study. **H**, **K**. IHC analysis of CDX tumor sections shows STIP1 knockdown reduces expression of proliferation marker Ki-67. Quantification of Ki-67 staining intensity is presented. **L**. PDX clinical information. **M-Q**. For PDXs, ESCC tumor fragments were implanted subcutaneously into immunodeficient mice. When PDX tumors reached 150-200 mm^3^, mice were randomized into treatment groups (n=8/group) for intratumoral injection with control virus or STIP1 shRNA lentiviruses every 3 days for 4 weeks. Tumor volumes and mouse weights were measured throughout. **O, R**. IHC staining of PDX tumors indicates STIP1 knockdown decreases Ki-67 levels. Data represent mean ± SD. **p* < 0.05, ***p* < 0.01 by Student’s unpaired t-test.

**
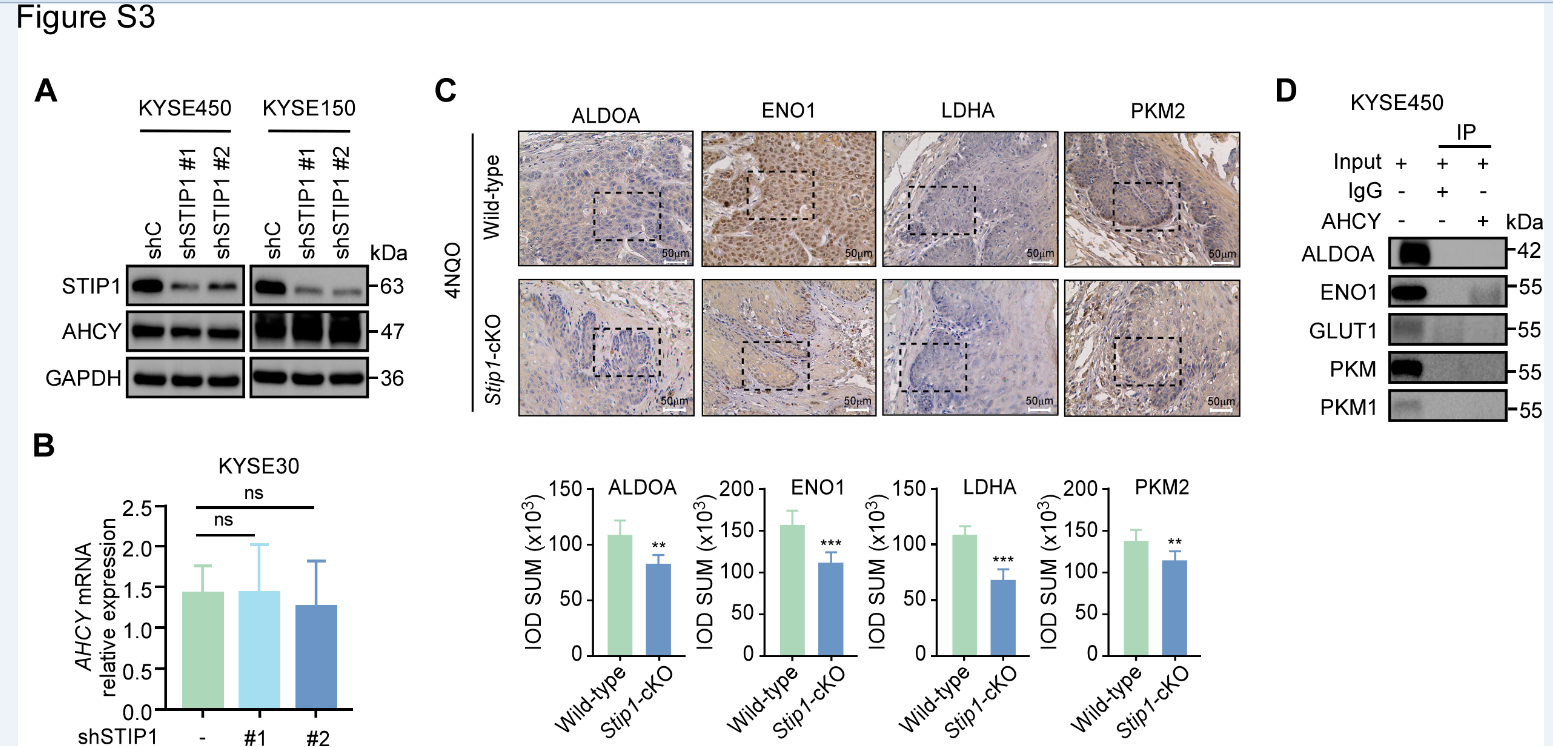
Supplemental Figure 3. STIP1 knockdown decreases AHCY protein level**

**A.** WB to check AHCY protein level after knockdown STIP1 in KYSE450 and KYSE150 cells. **B**. q-PCR to detect the mRNA level of AHCY after knockdown STIP1 in KYSE30 cells. **C**. IHC staining analysis of ALDOA, ENO1, LDHA and PKM2 expression in esophageal tissues and quantified protein expression levels, unpaired t-test, mean ± SD, ***p*<0.01, ****p*<0.001.

**
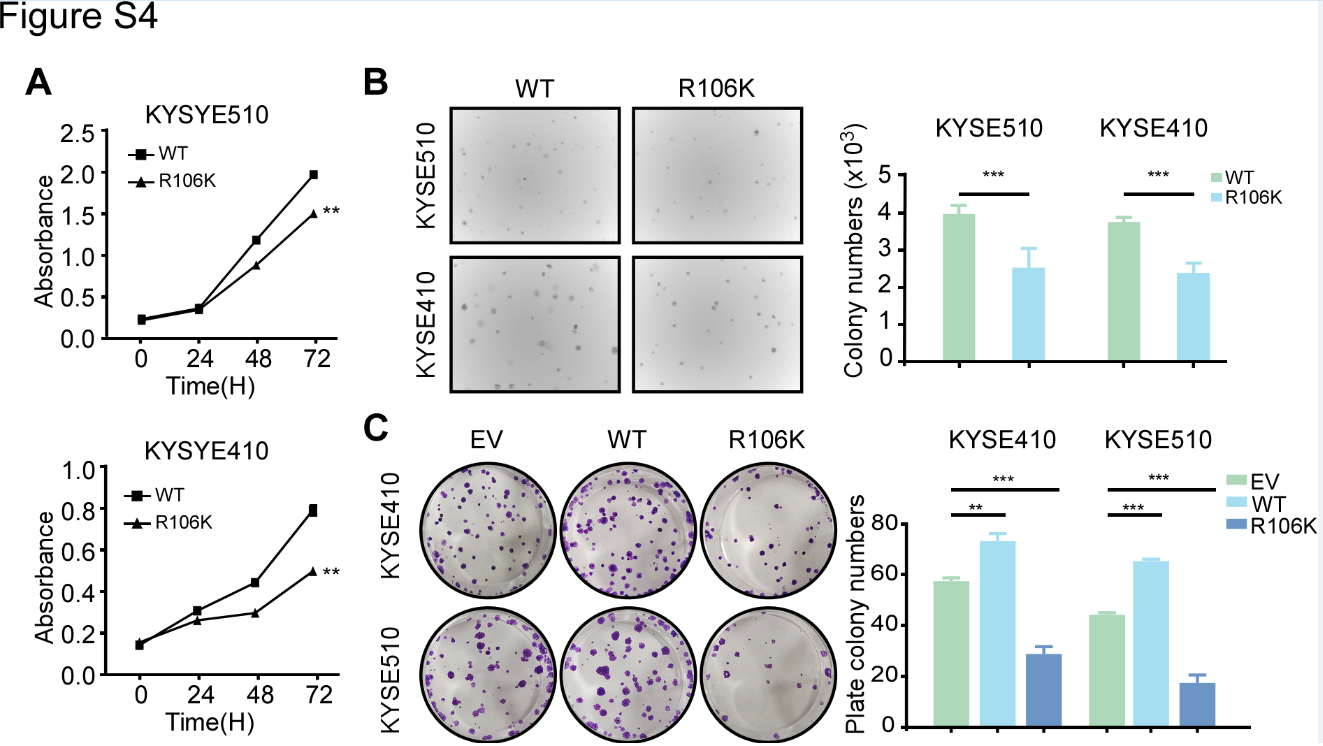
**

**Supplemental Figure 4**. **Blocking LDHA methylation at R106 residue inhibits tumor progression**

**A**. MTT assays show R106K mutation impairs ESCC cell proliferation. **B**. Soft agar colony formation is decreased with R106K mutant LDHA versus wildtype. Colonies numbers were calculated between the WT and mutant group in the right panel. **C**. R106K mutation reduces foci formation of ESCC cells. Plate colony numbers were analysis in WT and mutant groups in the right panel. Data are mean ± SD of 3 experiments. **p* < 0.05, ***p* < 0.01, ****p* < 0.001 by Student’s t-test.


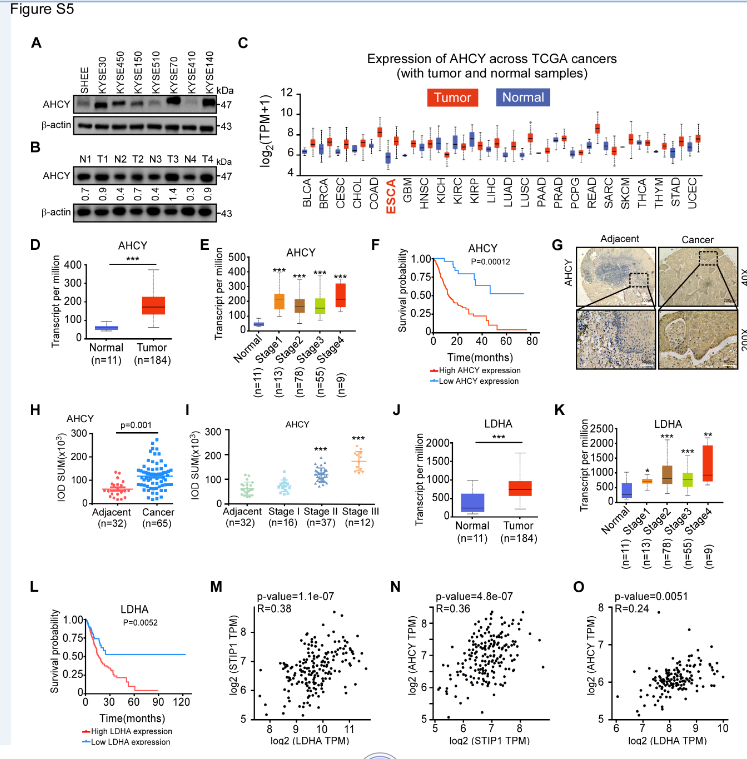


**Supplemental Figure 5**. **AHCY is highly expressed in ESCC and predicts the poor prognosis**

**A**. Western blot shows AHCY protein levels across a panel of ESCC cell lines versus normal esophageal epithelial cells. **B**. In 4 paired ESCC patient tissues, AHCY is upregulated in tumors compared to adjacent normal epithelium. **C**. Analysis of TCGA data shows transcriptional upregulation of AHCY in ESCC and other cancer types versus respective normal tissues. The TCGA database was used to evaluate the protein expression levels of AHCY in normal and cancer tissues (**D**), comparing expression across tumor stages(**E**), and investigating its potential prognostic significance(**F**). **G**. Representative immunohistochemical staining shows increased AHCY expression in ESCC tumors compared to adjacent normal epithelium. **H**. Quantitative analysis of IHC staining intensity reveals higher AHCY levels in unpaired ESCC tumors versus normal tissues. **I**. AHCY expression is increased in higher stage ESCC tumors. TCGA database was utilized to comprehensively evaluate LDHA expression, analyzing: expression levels in normal and cancerous tissues (**J**), LDHA expression profiles across tumor stages(**K**), and (3) correlations between LDHA expression and patient prognostic outcomes (**L**). The correlation analysis between STIP1, AHCY and LDHA were performed (**M-O**), Data are mean ± SD. **p* < 0.05, ***p* < 0.01, ****p* < 0.001 by Student’s t-test.

**
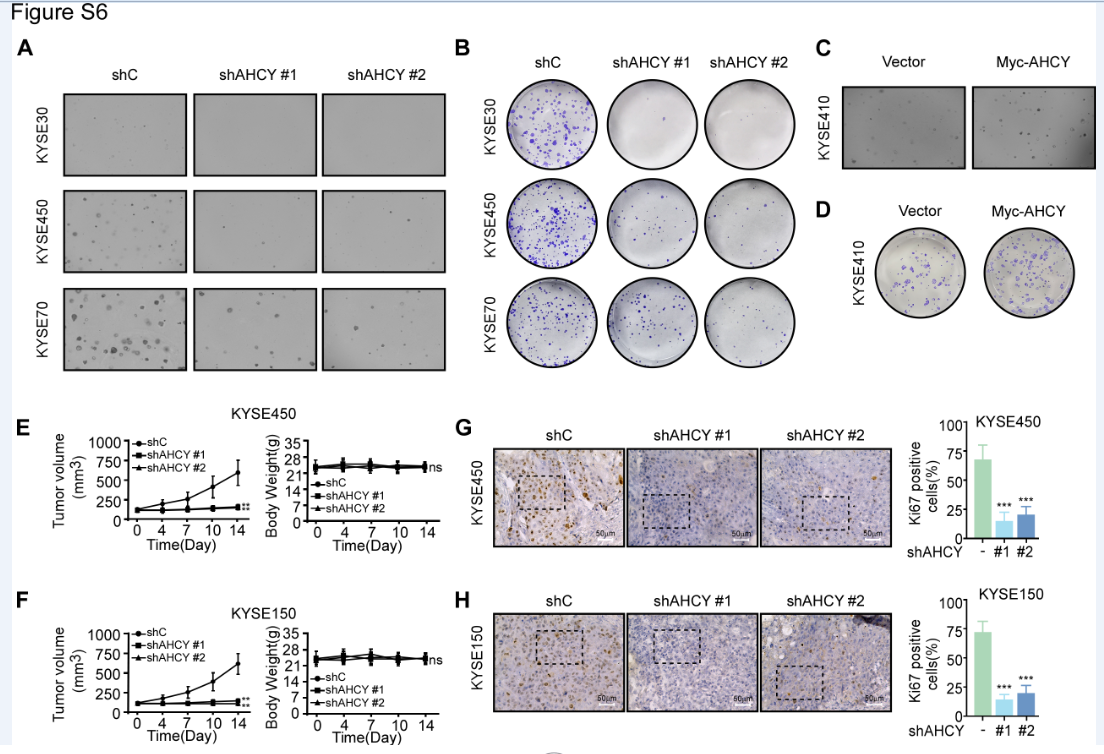
**

**Supplemental Figure 6.** **AHCY knockdown suppresses esophageal cancer tumor growth *in vitro* and *in vivo***

**A-B**. Soft agar and foci formation assays demonstrate AHCY knockdown reduces anchorage-independent growth and colony formation of ESCC cells. **C-D**. AHCY overexpression increases clonogenicity and foci formation of ESCC cells. **E-F**. In CDXs, ESCC cells expressing control shRNA or AHCY shRNAs were injected subcutaneously into athymic nude mice (n=8/group). Tumor volumes and mouse body were measured over time using calipers. **G-H**. IHC analysis of CDX tumor sections shows AHCY knockdown decreases expression of proliferation marker Ki-67. Quantification of Ki-67 staining intensity is presented. Data represent mean ± SD of triplicate experiments. **p* < 0.05, ***p* < 0.01, ****p* < 0.001 by Student’s unpaired t-test comparing AHCY knockdown versus control groups.


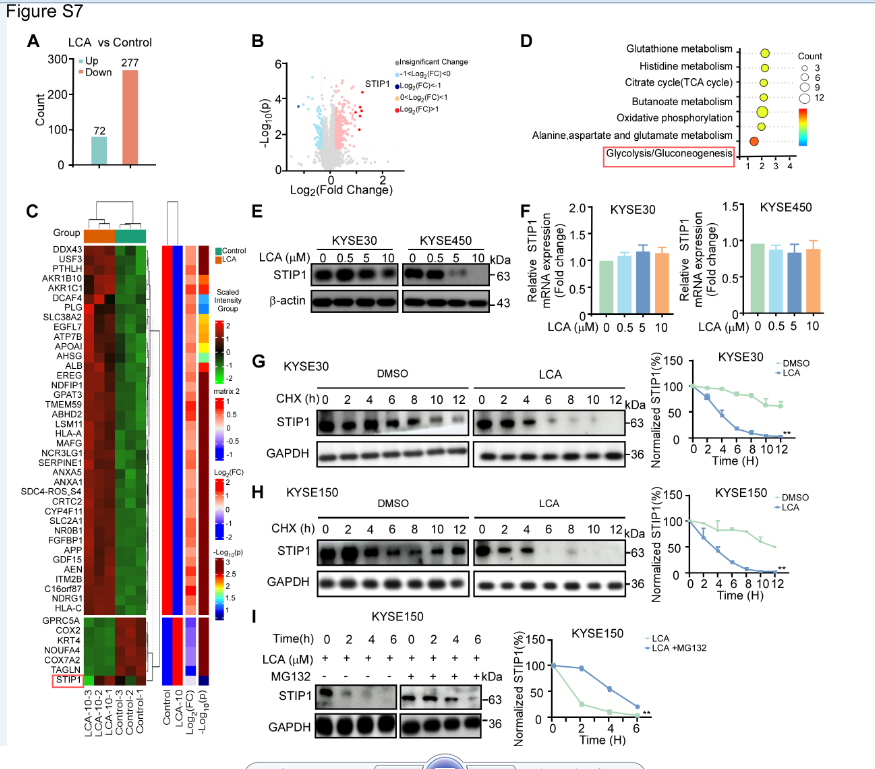


**Supplemental Figure 7. STIP1 as the target of LCA**

**A.** Following treatment with LCA, proteomic analysis revealed an upregulation of 72 proteins and a concomitant downregulation of 277 proteins. **B.** Volcano plots show differentially expressed proteins with ≥2-fold change cutoff. **C**. Heatmap shows relative expression of significantly altered proteins with LCA treatment. **D**. Pathway enrichment analysis reveals LCA impacts diverse metabolic processes including glycolysis. **E**. KYSE30 and KYSE450 cells treated with different doses of LCA, western blot was used to measured STIP1 expression. β-Action as the loading control. **F**. After treated with different doses of LCA, the STIP1 mRNA was detected my qPCR. **G-H**. Left panel: CHX (100nM) was added to KYSE30 and KYSE150 cells, and the cells were treated with DMSO or 10 µM LCA for the indicated times. **I**.KYSE150 was treated with 0.5 µM LCA and then incubated with MG132 or DMSO for the indicated times. and then STIP1 protein level was assessed by Western blotting. The STIP1 band intensities were quantified by Image Pro Plus software. Right panel: quantification of STIP1 expression normalized to GAPDH expression. Data are mean ± SD of 3 experiments. **p* < 0.05, ***p* < 0.01, by Student’s t-test.


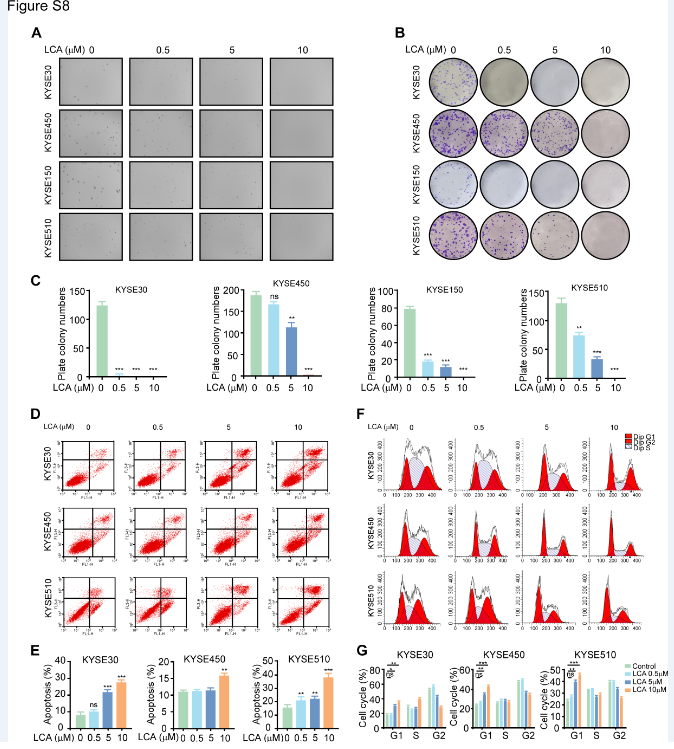


**Supplemental Figure 8. LCA inhibits cell proliferation and induces cell apoptosis**

**A.** Anchorage-independent growth and are decreased by LCA in a dose-dependent manner. Soft agar colony formation assays in ESCC cells treated with increasing concentrations of LCA. **B**. Foci formation assay was performed in ESCC cells treated with different concentration of LCA. **C**. Plate colony numbers were calculated after treated various doses of LCA. **D**. Apoptosis analysis by flow cytometry using Annexin V/PI staining demonstrates LCA increases the apoptotic cell population. **E.** Quantification of % apoptotic cells after varying LCA concentrations is shown. **F**. Cell cycle analysis by flow cytometry shows LCA induces G1 arrest. **G.** Quantification of cell cycle distribution after treatment with different LCA doses is presented. Data are mean ± SD of 3 experiments. **p* < 0.05, ***p* < 0.01, by Student’s t-test.


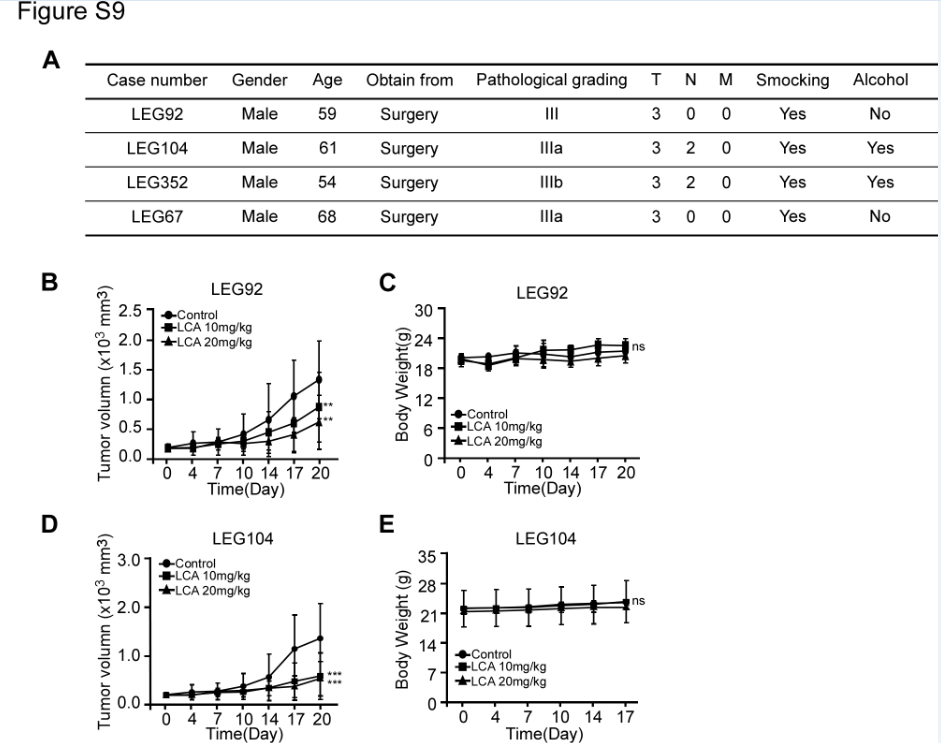


**Supplemental Figure 9. LCA suppressed tumor growth in ESCC PDX models**

**A.** The clinical information of PDX models. **B-****E**. ESCC patient-derived xenograft (PDX) bearing mice were treated with LCA (10 or 20 mg/kg) or vehicle control by daily oral gavage for 8 weeks (n=8/group). Tumor volumes and mouse body weights were measured twice weekly by calipers. Data represent mean ± SD. **p* < 0.05, ***p* < 0.01, ****p* < 0.001 by Student’s t-test comparing LCA treatment versus control.
