# Supplementary material for: STIP1 drives Metabolic Reprogramming in Esophageal Squamous Cell Carcinoma via AHCY‐LDHA Axis
Source: Exploration (Beijing). 2025 May 25;5(5):20240198. doi: 10.1002/EXP.20240198 (PMC12561186; doi:10.1002/EXP.20240198)
Supplement: Supplementary file 2 — Supporting Information [file EXP2-5-20240198-s001.docx]

**Supplement Table 1: PCR Primers**

**PCR Primer 1 :**

F1: 5’-AACATTGGTGGGGACTAACTCTAC-3’

R1: 5’-AGAGAAATGAAGGCTTGGAGAGATG-3’

**PCR Primer 2:**

F1: 5’-AACATTGGTGGGGACTAACTCTAC-3’

R2: 5’-ACAGGGCCCTAATTAAATGTCTCA-3’

Homozygotes: one band with 585 bp

Heterozygotes: two bands with 585 bp and 533 bp

Wildtype allele: two bands with 533 bp

| Antibody | [Manufacturer](javascript:;) | Lot number | Application | Dilution |
| --- | --- | --- | --- | --- |
| STIP1 | Abcam | ab126724 | WB; IHC | 1:1000; 1:250 |
| PKM | Abcam | ab89364 | WB | 1:1000 |
| ENO1 | Abcam | ab227978 | WB | 1:1000 |
| AHCY | Proteintech | 10757-2-AP | WB; IP; IF; IHC | 1:1000; 1:100; 1:100; 1:100 |
| STIP1 | Proteintech | 68155-1-Ig | IF; IP | 1:200; 1:100 |
| LDHA | Proteintech | 19987-1-AP | WB | 1:1000 |
| ALDOA | Proteintech | 17217-1-AP | WB | 1:1000 |
| PKM2 | CST | #4053 | WB | 1:1000 |
| CDK4 | CST | #12790 | WB | 1:1000 |
| Cyclin D | CST | #2978 | WB | 1:1000 |
| Bax | CST | #5023 | WB; IHC | 1:1000; 1:200 |
| Caspase 3 | CST | #9662 | WB | 1:1000 |
| Cleaved Caspase 3 | CST | #9664 | WB | 1:1000 |
| PARP | CST | #9542 | WB | 1:1000 |
| Cleaved PARP | CST | #5625 | WB | 1:1000 |
| PRMT3 | Invitrogen | 730020 | WB | 1:1000 |
| anti-Flag | Sigma-Aldrich | #F1804 | WB; IP | 1:1000; 1:200 |
| PKM2 | CST | #4053 | WB | 1:1000 |
| GAPDH | CST | #97166 | WB | 1:3000 |
| Beta actin | Proteintech | HRP-60008 | WB | 1:1000 |
| Ki67 |  | Ab21700 | IHC | 1:100 |
| Anti-Myc | abcam | Ab32 | WB; IP | 1:1000; 1:200 |
| Anti-HA | abcam | Ab9110 | WB | 1:1000 |
| Ubiquitin | Proteintech | 10201-2-AP | WB | 1:1000 |
| LDHAme1 | CST |  | WB | 1:1000 |
| LDHAme2 | CST |  | WB | 1:1000 |
| LDHA R106me1 | CST |  | WB | 1:1000 |
| LDHA R106me2 | CST |  | WB | 1:1000 |
| Normal Rabbit IgG | CST | #2729 | IP | 1:100 |
| Alexa Fluor 488-labeled goat anti-mouse IgG | Invitrogen | A11029 | IF | 1:200 |
| Alexa Fluor 568-labeled donkey anti-rabbit IgG | Invitrogen | A10042 | IF | 1:200 |

**Supplemental Table 2: Antibody list**
